# A comparative analysis of fruit fly and human glutamate dehydrogenases in *Drosophila melanogaster* sperm development

**DOI:** 10.3389/fcell.2023.1281487

**Published:** 2023-11-02

**Authors:** Viktor Vedelek, Balázs Vedelek, Péter Lőrincz, Gábor Juhász, Rita Sinka

**Affiliations:** ^1^ Department of Genetics, University of Szeged, Szeged, Hungary; ^2^ Hungarian Research Network, Biological Research Centre, Developmental Genetics Unit, Szeged, Hungary; ^3^ Department of Anatomy, Cell and Developmental Biology, Eötvös Loránd University, Budapest, Hungary; ^4^ Hungarian Research Network, Biological Research Centre, Institute of Genetics, Szeged, Hungary

**Keywords:** *Drosophila*, GLUD1, GLUD2, glutamate dehydrogenase, Bb8, testis, spermatogenesis, GDH

## Abstract

Glutamate dehydrogenases are enzymes that take part in both amino acid and energy metabolism. Their role is clear in many biological processes, from neuronal function to cancer development. The putative testis-specific *Drosophila* glutamate dehydrogenase, Bb8, is required for male fertility and the development of mitochondrial derivatives in spermatids. Testis-specific genes are less conserved and could gain new functions, thus raising a question whether Bb8 has retained its original enzymatic activity. We show that while Bb8 displays glutamate dehydrogenase activity, there are significant functional differences between the housekeeping Gdh and the testis-specific Bb8. Both human GLUD1 and GLUD2 can rescue the *bb8* ^*ms*^ mutant phenotype, with superior performance by GLUD2. We also tested the role of three conserved amino acids observed in both Bb8 and GLUD2 in Gdh mutants, which showed their importance in the glutamate dehydrogenase function. The findings of our study indicate that *Drosophila* Bb8 and human GLUD2 could be novel examples of convergent molecular evolution. Furthermore, we investigated the importance of glutamate levels in mitochondrial homeostasis during spermatogenesis by ectopic expression of the mitochondrial glutamate transporter Aralar1, which caused mitochondrial abnormalities in fly spermatids. The data presented in our study offer evidence supporting the significant involvement of glutamate metabolism in sperm development.

## Introduction

*Drosophila melanogaster* spermatogenesis results in approximately 1.8-mm-long sperm cells. To achieve this length, drastic changes in the structure and quality are required. Mitochondria are crucial elements of sperm production (Varuzhanyan and Chan, 2020; Vertika et al., 2020; Park and Pang, 2021). In *Drosophila*, mitochondrial differentiation is spectacular during the post-meiotic development of spermatids (Fuller et al., 1993; White-Cooper and Henderson, 2004). Right after meiosis, the mitochondria aggregate and form the nebenkern structure, which reaches the size of the haploid nucleus. As the round spermatids start to elongate, the nebenkern unfurls and two mitochondrial derivatives emerge (Fuller et al., 1993). During spermatid elongation, mitochondrial derivatives serve as a platform for microtubules to drive the elongation process as they run along the tail region (Noguchi et al., 2011). The post-meiotic development has multiple presumably energy-demanding synthetic and reorganization processes. Approximately 70% of the sperm membranes are produced in these stages, and the testis-specific proteins like paracrystalline material and protamines need to be synthesized (Tokuyasu, 1975; Noguchi et al., 2011; Barckmann et al., 2013; Steinhauer, 2015; Laurinyecz et al., 2019a). In addition to nuclear remodeling and the histone-to-protamine transition, considerable cytoskeletal reorganization occurs during elongation and individualization. Therefore, an interesting question is raised: how do spermatids address the energy requirements of their development? One potential way is to utilize different metabolites as an additional energy source. There are known examples of how cells can utilize additional resources. In mammals, the developing spermatids mostly feed on lactate (Boussouar and Benahmed, 2004). It is known that glutamine could also serve as a potent energy source (glutaminolysis) for many cell types under physiological circumstances, like cell divisions and immune activity, or under pathological circumstances, like carcinogenesis (Newsholme et al., 1985; Newsholme, 2001; Newsholme et al., 2003; Mastorodemos et al., 2005; Scalise et al., 2017; Yang et al., 2017). During glutaminolysis, glutamine is transformed into glutamate and eventually alpha-ketoglutarate. The main component at this crucial metabolic branch point between the central energy metabolism and amino acid metabolism is the glutamate dehydrogenase enzyme (GDH) (EC 1.4.1.2) (Smith et al., 2019). GDH catalyzes the reversible reaction of the deamination of glutamate to alpha-ketoglutarate (alternatively 2-oxoglutarate). This reaction is a link between the TCA cycle (tricarboxylic acid cycle) and amino acid metabolism: alpha-ketoglutarate is part of the TCA cycle, and amino acid glutamate is necessary for the production of glutamine, histidine, proline, and arginine. The benefit of the catabolism of glutamate to alpha-ketoglutarate is that it makes it possible to directly utilize alpha-ketoglutarate in central energy metabolism, and it produces ammonia, which could be used for amino acid synthesis. Glutamate has a wide variety of biological functions; it serves as a neurotransmitter and is necessary for GABA and glutathione synthesis. The importance of glutamate metabolism is also traceable under pathologic conditions like cancer, where glutamate dehydrogenases were suggested to play multiple roles. On one hand, they participate in glutaminolysis, which boosts the metabolic activity of the cancer cells; on the other hand, in metastatic cancer cells, they participate in the reverse glutamate dehydrogenase activity, which helps fixate ammonium and promote cell proliferation (Altman et al., 2016; Scalise et al., 2017; Spinelli et al., 2017; Moreno-Sánchez et al., 2020; Shao et al., 2021). GDH also plays a role in insulin secretion and related diseases (Stanley et al., 1998; Macmullen et al., 2001; Li et al., 2010; Smith et al., 2019; Luczkowska et al., 2020).

In animals, glutamate dehydrogenases consist of a NAD(H)-binding domain, a dimerization domain, a hinge or pivot helix, and an antenna region (Benachenhou-Lahfa et al., 1993; Peterson and Smith, 1999; Smith et al., 2001; Andersson and Roger, 2003; Banerjee et al., 2003). According to the crystal structures of the bovine GDH, GDHs could have different levels of polymerization. GDH monomers form a homotrimer by their antenna regions, two trimers could be assembled into a hexamer, and a filament may be formed from the hexameric units (Munn, 1972; Peterson and Smith, 1999; Banerjee et al., 2003) (Supplementary Figure S1A). The comparative analyses of glutamate dehydrogenases from different organisms shed light on the increasing complexity of enzymatic function and regulation (Banerjee et al., 2003; Bunik et al., 2016). The glutamate dehydrogenase activity is influenced by a wide variety of compounds. The most important compounds are GTP and NADH, which have an inhibitory effect, while ADP and leucine have an activator effect on mammalian GDH (Smith and Stanley, 2008; Li et al., 2012; Li et al., 2014; Bera et al., 2020). Available 3D structures help understand the allosteric regulation of the enzyme by these regulators as their binding pockets were identified and opened and closed forms of GDHs were determined (Peterson and Smith, 1999; Smith et al., 2001; Smith and Stanley, 2008; Li et al., 2012; Li et al., 2014; Borgnia et al., 2016; Tomita et al., 2017; Dimovasili et al., 2021).

In humans, there are two glutamate dehydrogenases; GLUD1 has a ubiquitous expression pattern, while the evolutionarily younger GLUD2 is enriched in the testis, brain, and retina (Shashidharan et al., 1994; Spanaki et al., 2010; Spanaki et al., 2015). GLUD1 and GLUD2 have 90% sequence homology; however, their enzymatic regulation is different (Bunik et al., 2016). GLUD2 basal activity is lower than that of GLUD1; however, activators l-leucine or ADP have a higher effect on its activity, while GLUD2 sensitivity to GTP is minimal (Plaitakis and Zaganas, 2001; Plaitakis et al., 2011; Shashidharan and Plaitakis, 2014). This difference in allosteric regulation led to significant evolutionary consequences. GTP serves as an indication of the cells’ energy state, and the high abundance of GTP inhibits the catabolic processes. The usage of glutamate to feed the TCA cycle is not energetically favorable when the sugar metabolism pathway could supply it. The GLUD2 enzyme’s capacity for glutamate catabolism could be advantageous in specialized cells, such as neurons. Neuronal expression of GLUD2 enables the efficient removal of excess glutamate neurotransmitters, thereby enhancing the neurotransmission efficacy (Spanaki et al., 2010). The role of GLUD2 is described in Parkinson’s disease and *IDH1* ^*R132H*^ gliomas (Chen et al., 2014; Zhang et al., 2020).

Interestingly, *Drosophila* also encodes two glutamate dehydrogenases, *gdh* and *bb8*. *gdh* has a ubiquitous expression, while *bb8* has a testis-enriched expression pattern (Vedelek et al., 2016; Vedelek et al., 2018). We previously reported that Bb8 is necessary for normal mitochondrial development in the post-meiotic development of spermatids. Lack of Bb8 resulted in the improper elongation of cysts, megamitochondria formation, and a problem with paracrystalline material accumulation in the mitochondrial derivatives of spermatids (Vedelek et al., 2016). Bb8 was also found in the SDS-resistant fraction of *Drosophila* sperm, which represents the paracrystalline material of the major mitochondrial derivative, suggesting a structural role for Bb8 (Laurinyecz et al., 2019b). The previously observed phenotypes in paracrystalline accumulation defects and the presence of Bb8 in the paracrystalline material suggest that Bb8 plays a structural role (Vedelek et al., 2016; Laurinyecz et al., 2019b).

In this study, we initially aimed to test the comparative conservation of glutamate dehydrogenases during spermatogenesis. The existence of testis-specific genes suggests the potential for the acquisition of novel functions, which raises concerns regarding the retention of enzymatic activity in Bb8. We demonstrated that Bb8 does possess glutamate dehydrogenase activity in the testis; however, functional disparities exist between the generally expressed Gdh and the testis-specific Bb8. Due to the similarities in tissue specificity of human GLUD2 and bb8, we tested GLUD1 and GLUD2 activities and functions during *Drosophila* spermatogenesis. Our investigation reveals that both human glutamate dehydrogenases (GLUD1 and GLUD2) can rescue the mutant phenotype of *bb8* ^*ms*^; however, the tissue-specific GLUD2 exhibits superior performance compared to GLUD1.

## Results

### Glutamate dehydrogenase phylogeny and structure

To better understand the differences and similarities between the somatic and tissue-specific glutamate dehydrogenases, we analyzed the protein sequences of human and *Drosophila* enzymes. Based on sequence alignments, *Drosophila* Bb8 is considerably different compared to *Drosophila* Gdh and human glutamate dehydrogenases (Vedelek et al., 2016). Bb8 has a 44% protein sequence similarity with human GLUD1 and GLUD2, and a 45% similarity with Gdh; meanwhile, Gdh has a 64% sequence similarity with GLUD1 and GLUD2 (Supplementary Figure S2). We also investigated the synonymous and non-synonymous mutations in Bb8 and Gdh in *Drosophila* species with SNAP analyses (Korber et al., 2000). We found that both Gdh and Bb8 sequences are under stabilizing selection, but Gdh-like genes are more conserved and contain fewer mutations (Supplementary Table S1A,B, Supplementary Figure S3). Investigating the substitutions in the predicted pockets, we found that Gdh-like sequences have fewer substitutions; meanwhile, Bb8-like sequences have a higher rate of synonymous and non-synonymous substitutions (Supplementary Table S1C), suggesting that Bb8 is less conserved and shows higher variability in *Drosophila* species. These results are in line with the previous phylogenetic analyses of *Drosophila* glutamate dehydrogenases (Vedelek et al., 2016). To further see how Bb8 differs from other Gdhs, we conducted a phylogenetic analysis on duplicated glutamate dehydrogenases in the Metazoa taxon (Supplementary Figure S3). We concluded that Bb8-like glutamate dehydrogenases are on a different branch than Gdh-like glutamate dehydrogenases. The Bb8-like branch is characteristic of flies and mosquitoes (Culicidae). *Drosophila* Gdh and Bb8 have a relatively large distance between them; compared to this, mammalian glutamate dehydrogenases are considerably more conserved, and the human glutamate dehydrogenases (GLUD1 and GLUD2) are much closer to each other, as expected. The phylogenic tree shows many vertebrate species that have multiple glutamate dehydrogenases, and these glutamate dehydrogenases seem to be less diverse in general; however, birds and reptiles have a more distant branch as well.

There is no crystal structure information on *Drosophila* Gdh and Bb8; however, similarity and machine-learning-based modeling (AlphaFold) provide models with high confidence (Jumper et al., 2021). These models revealed that despite the differences in protein sequences, *D. melanogaster* Gdh, Bb8, and mammalian glutamate dehydrogenases (bovine GDH) most likely have similar structures and conserved folds. Further homology-based complex models (InterEvDock3) of Gdh and Bb8 suggest similar tertiary structures and intermolecular organization of mammalian GDH as well (Supplementary Figure S1B, Supplementary Table S1D) (Quignot et al., 2021). Bb8 likely has the canonical animal glutamate dehydrogenase structure; it has a NAD-binding domain, a dimerization domain, a hinge or pivot helix, and an antenna domain (Peterson and Smith, 1999; Banerjee et al., 2003).

On the ClustalW-aligned human and *Drosophila* glutamate dehydrogenase protein sequences, the amino acids of the alpha-ketoglutarate-binding pocket, the GTP-binding pocket, the NAD-binding pocket, and the ADP-binding pockets were highlighted (Banerjee et al., 2003) (Supplementary Figure S2). Despite the relatively low sequence similarities with other GDHs, in its alpha-ketoglutarate-binding pocket (active center), Bb8 has the previously described conserved amino acids that are characteristic of glutamate dehydrogenases. However, the conserved amino acids in regulatory domains are more diverse: 8 out of 13 in the NAD-binding pocket, 13 out of 24 in the ADP-binding pocket(s), and 4 out of 14 amino acids in the GTP-binding pocket are identical with the human GLUD1 amino acids (Supplementary Figure S2, Supplementary Figure S4). Interestingly, the conserved regulatory NAD-binding pocket of Bb8 shows a higher amino acid sequence similarity to GLUD2. Otherwise, the ADP- and GTP-binding pockets are much less conserved in Bb8 compared to Gdh or human GLUDs, which raises the possibility of the alternative regulation of its enzymatic activity. We used the Bb8 AlphaFold model to visualize the amino acids of the conserved sites of the Bb8 protein, which represents the topology of the binding pockets of the enzyme (Supplementary Figure S4).

**FIGURE 1:**
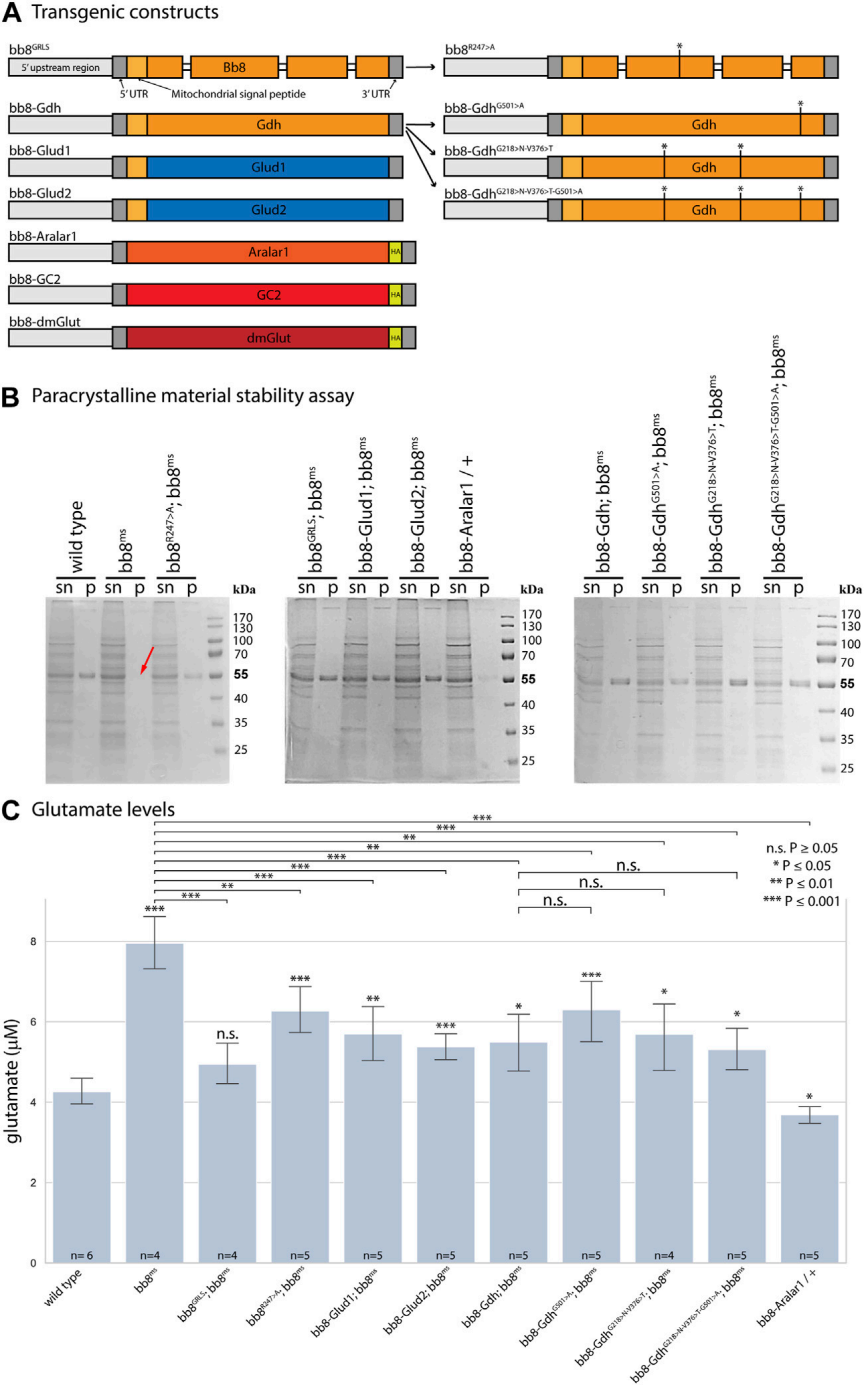
Enzymatic and structural properties of glutamate dehydrogenases. **(A)** Schematic representation of transgenic constructs established for this study. Thick bars represent exons, *Drosophila* genes are marked with orange–red hues, and human sequences are marked blue. **(B)** In paracrystalline material stability assays, ‘sn’ stands for supernatant, and ‘p’ stands for pellet. The red arrow shows missing SDS-resistant fractions (∼55 kDa) in *bb8ms* mutant samples. **(C)** Summary of glutamate assays. Significant differences compared to the wild-type are marked. The number of biological replicates is presented at the base of each column. Each biological replicate was produced from 20 flies.

Based on the structural similarities, we can hypothesize that Bb8 can be capable of forming similar filamentous and lamellar structures like mammalian glutamate dehydrogenases (Munn, 1972; Banerjee et al., 2003) (Supplementary Figure S1). This structure could be incorporated into the paracrystalline material of the major mitochondrial derivative of the spermatids. The paracrystalline material primarily consists of sperm leucyl aminopeptidase (S-Lap) proteins. The S-Lap family members are essential for paracrystalline material formation; however, their enzymatic activity is lost (Laurinyecz et al., 2019b). We earlier showed the presence of the Bb8 protein in the paracrystalline material of the major mitochondrial derivative by mass spectrometry, and electron micrographs of *bb8* ^*ms*^ mutant testis also showed paracrystalline material accumulation defects (Vedelek et al., 2016; Laurinyecz et al., 2019b). The biochemical resistance of the paracrystalline material in *bb8* ^*ms*^ mutants to SDS has not been previously examined. Consequently, we performed a stability assay on the paracrystalline material, following the methodology outlined by Laurinyecz et al. in 2019. This involved isolating the SDS-resistant fraction from protein extracts obtained from the testis (Figure 1B) (Laurinyecz et al., 2019b). In *bb8* ^*ms*^ mutants, the SDS-resistant fraction is not detectable (Figure 1B), which further points to the importance of Bb8 in paracrystalline material formation. Despite these observations, Bb8 enzymatic function can influence paracrystalline material formation: the previously presented megamitochondria occurrence in S2 cells was linked to abnormal glutamate homeostasis (Shim et al., 2009; Shim et al., 2011); therefore, the swelling of mitochondria due to metabolic abnormalities could easily disturb the normal synthesis of the paracrystalline material.

### Glutamate dehydrogenase activity in spermatids

**FIGURE 2:**
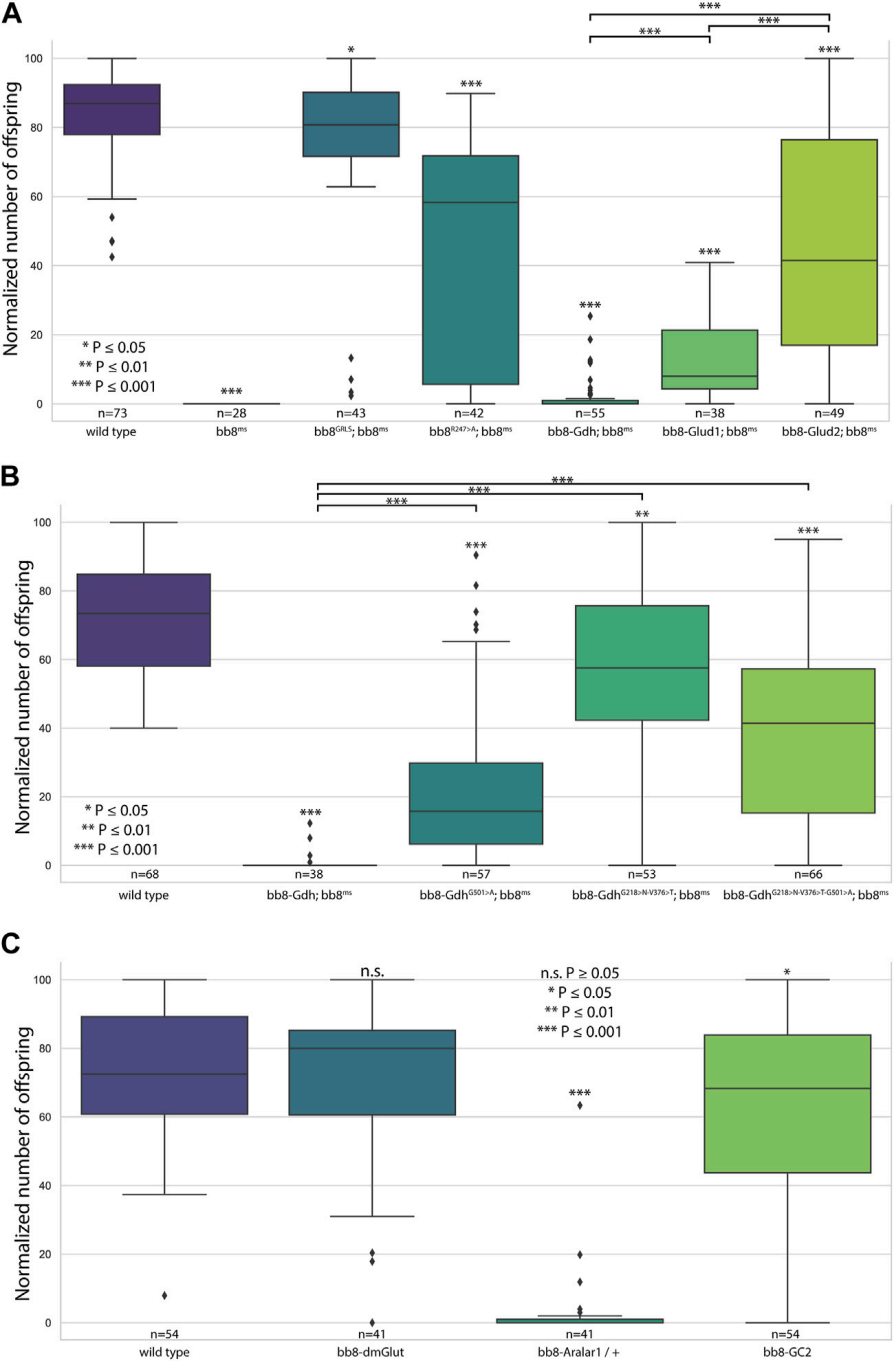
Fertility assays. Boxplots represent the normalized offspring count of **(A)** glutamate dehydrogenase rescue constructs on the *bb8ms* background, **(B)** *Drosophila* Bb8-Gdh variant rescue on the *bb8ms* background, and **(C)** Bb8-driven transgenes on the wild-type background. Significant differences compared to wild-type are marked on top of each box, and additional significance values are represented above the connected boxes. The number of individual males in fertility tests is marked above the genotypes. **(A)** *nvirgins/cross* = 5; **(B, C)** *nvirgins/cross* = 3.

Since the Bb8 enzymatic function was not tested previously, we investigated it indirectly by measuring the glutamate levels in *bb8* ^*ms*^ mutant testes with a fluorimetric assay (Figure 1C). In *bb8* ^*ms*^ mutant testes, the glutamate levels were elevated, which could be explained by the absence of Bb8 glutamate dehydrogenase activity. This is consistent with the formation of megamitochondria in S2 cells, which was also associated with elevated glutamine and glutamate levels (Shim et al., 2009; Shim et al., 2011). Vedelek et al. previously reported the presence of a peculiar mitochondrial phenotype, characterized by megamitochondria formation, in *bb8* ^*ms*^ spermatids. This finding also hinted at a possible enzymatic role for Bb8. The *Drosophila* testis, along with the *bb8* ^*ms*^ mutant, serves as a valuable model for investigating the potential impact of glutamate metabolism and GDH enzyme function. The homozygous *bb8* ^*ms*^ mutant flies are viable, but males are completely sterile (Figure 2A). The mutation causes a spectacular and easy-to-follow phenotype: in every elongating cyst, megamitochondria formation occurs, which is visible even by phase-contrast microscopy (Supplementary Figure S5A, B). The mitochondrial abnormalities can be visualized further by membrane potential-sensitive MitoTracker staining or by immunostaining of the mitochondrial Complex V subunit bellwether with the α-atp5α antibody (Supplementary Figure S6A, B, Supplementary Figure S7A, B). The *bb8* ^*ms*^ mutant cysts are not fully elongated, but the axoneme is matured, which can be visualized and measured using the AXO49 antibody (Supplementary Figure S8A,B,N). At the end of elongation, actin cones form around the elongated nucleus, establishing the individualization complex (IC), which migrates toward the basal end of the cyst. The process results in sperm bundles and a waste bag. The individualization process of the elongated spermatids is severely damaged in *bb8* ^*ms*^ mutants, and even actin cone formation is abnormal, which was investigated by phalloidin staining (Supplementary Figure S9A, A’, B, B’, N). Overall, *bb8* ^*ms*^ offers a good background and set of observable phenotypes to investigate glutamate metabolism.

**FIGURE 3:**
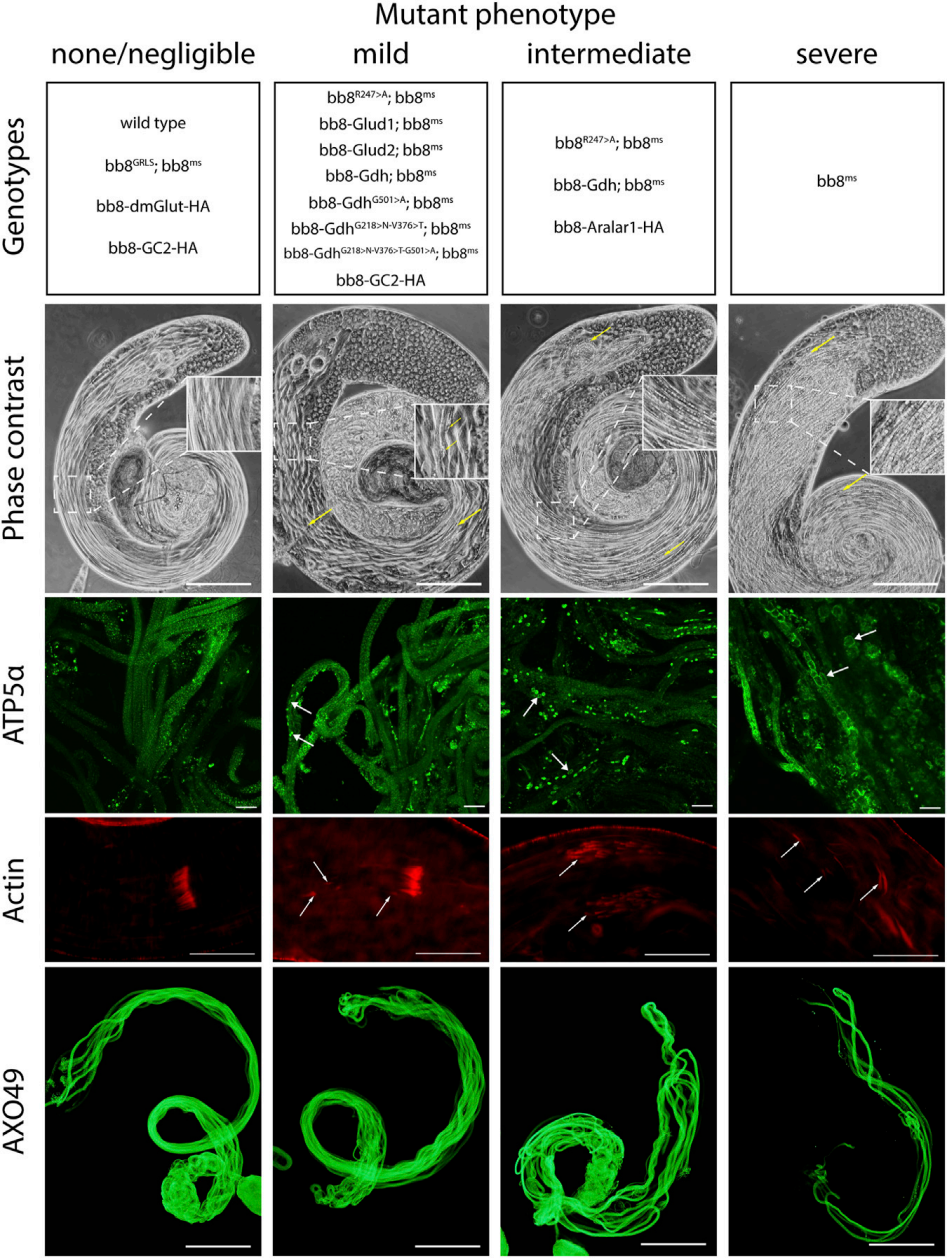
Summary of phenotype classes based on light microscopic examinations. Some of the genotypes are present in multiple categories. Representative images for each genotype can be found in Supplementary Figure S5–S9. Arrows point to abnormalities. Scale bars represent phase contrast, AXO49–200 μm, ATP5α–20 μm, and phalloidin (actin) –50 μm.

**FIGURE 4:**
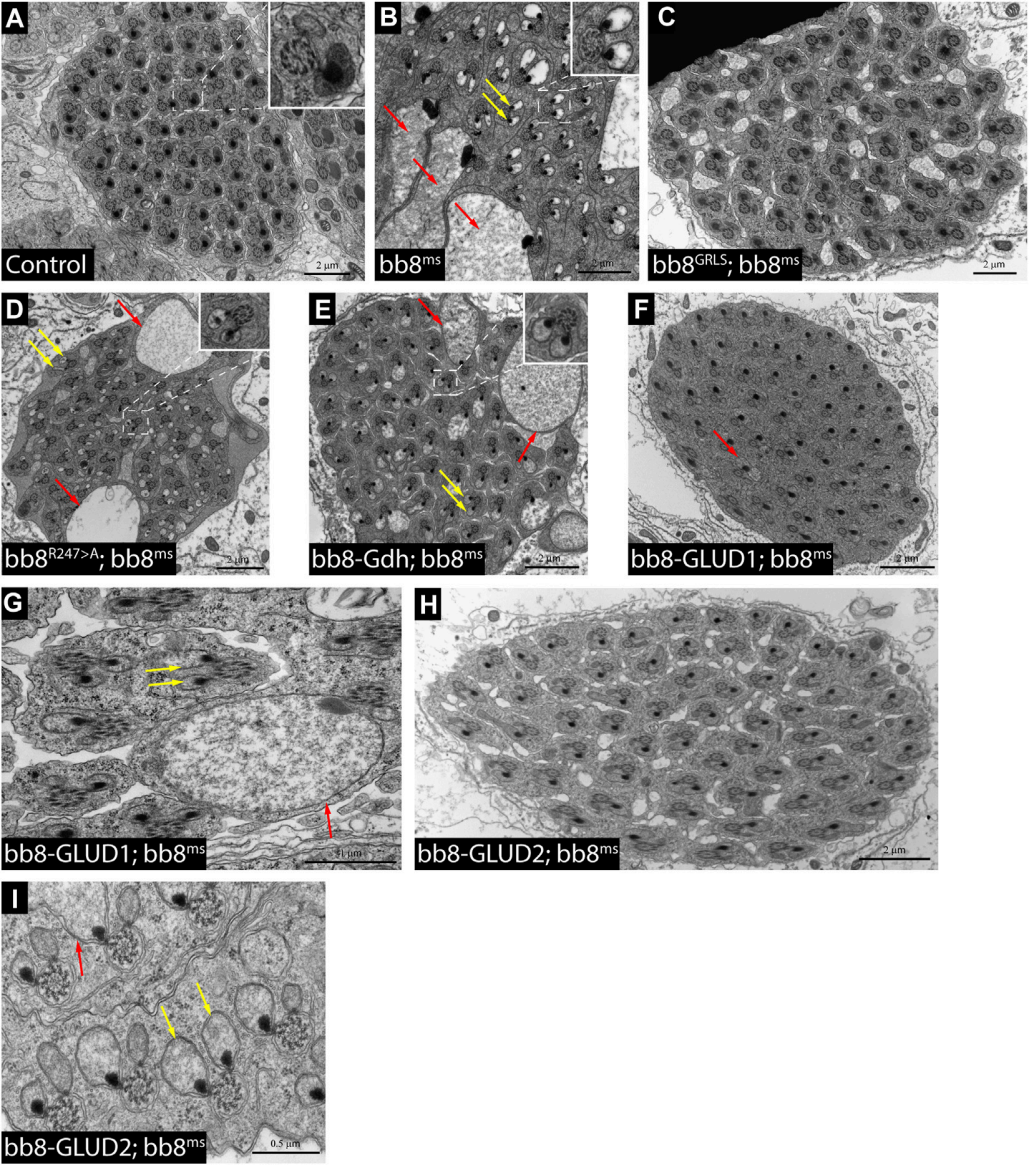
Transmission electron micrograms of testis cross sections representing elongating cysts. Red arrows point to swollen mitochondrial structures, and yellow arrows highlight spermatids where paracrystalline material accumulates in both mitochondrial derivatives. The length values are presented by the scale bars positioned above them.

To further investigate the role of glutamate dehydrogenase activity, we designed a mutation to inhibit the enzymatic activity of Bb8 by introducing an amino acid change to position 247 (272 on alignment), arginine to alanine (Bb8^R247>A^) (Figure 1A, Supplementary Figure S2, Supplementary Figure S4A). This position is shared between the alpha-ketoglutarate-binding pocket and the NAD-binding pocket of Bb8. We expected the loss of enzymatic activity while we kept the structural integrity of the enzyme. To achieve this, first, we established a wild-type genomic rescue construct as the control (*bb8* ^*GRLS*^), which contains the entire bb8 gene and ∼500-bp upstream regulatory region. We used this construct to introduce amino acid changes in the enzymatic active centrum (Bb8^R247>A^). We investigated the transgenes’ rescuing capacity over the *bb8* ^*ms*^ mutant background (Figure 1; Figure 2A; Figure 3, Supplementary Figure S5–S9C, D, C′, D′; Figure 4C,D). The *bb8* ^*GRLS*^ *; bb8* ^*ms*^ flies rescue the *bb8* ^*ms*^ mutant phenotype in every aspect (Figure 1; Figure 2A; Figure 3, Supplementary Figure S5–S9C, C′; Figure 4C). Surprisingly, we observed a partial rescue of the male sterile phenotype of the *bb8* ^*R247*>A^ mutants (Figure 2A). Male species of the *bb8* ^*R247*>A^; *bb8* ^*ms*^ flies showed megamitochondria formation (Figure 3, Supplementary Figure S5–S7D, D′; Figure 4D). The glutamate levels in these flies were lower than that in the *bb8* ^*ms*^ mutant’s testes (Figure 1C). In contrast to *bb8* ^*ms*^, the cysts elongate properly, and individualization complexes are formed; however, disturbed individualization complexes are also present in *bb8* ^*R247*>A^; *bb8* ^*ms*^ flies (Supplementary Figure S8D,N, S9D,N,D′). In this line, we also found the formation of the SDS-resistant paracrystalline material; however, paracrystalline material abnormalities can be observed in TEM images (Figure 1B; Figure 4D). We concluded that the *bb8* ^*R247*>A^; *bb8* ^*ms*^ allele is hypomorphic, and the altered Bb8^R247>A^ is partially active; hence, the observed phenotype is weaker compared to the *bb8* ^*ms*^ phenotype. These results suggest that the wild-type Bb8 enzyme activity is necessary for proper spermatid development.

Next, we investigated the general glutamate dehydrogenase function in spermatids. To see the potential functional differences between Gdh and Bb8, we established chimeric glutamate dehydrogenases (Figure 1A). We prioritized the correct expression pattern and mitochondrial localization; therefore, using *in silico* methods, we determined the mitochondrial signal peptides of Bb8. Gdh, GLUD1, and GLUD2 were cloned using the Bb8 genomic regions (including ∼500 bp 5′ and 3′ UTR regions) and with the Bb8 mitochondrial signal peptide (*bb8-Gdh*, *bb8-GLUD1*, and *bb8-GLUD2*) (Figure 1A). We investigated the rescue capacity of these constructs on a *bb8* ^*ms*^ mutant background. Based on the number of offspring, we observed the best rescuing capacity with the genomic rescue *bb8* ^*GRLS*^ construct, followed by *bb8-GLUD2*, *bb8-GLUD1*, and *bb8-Gdh* (Figure 2A). All the transgenic constructs were able to partially rescue the *bb8* ^*ms*^ sterility (Figure 2A). Megamitochondria formation is not characteristic for every cyst in the mutant (Figure 3, Supplementary Figure S5–S7A–C, E–G,A′C′,E′–G′; Figures 4C–I), and cyst elongation (Supplementary Figure S8B,C,E–G, N) and individualization complexes were properly formed; however, we observed disturbed migrating individualization complexes (Supplementary Figure S5–S9A–C, E–G,A′C′,E′–G′). Every rescue construct has lower glutamate accumulation based on the fluorimetric measurements compared to the *bb8* ^*ms*^ mutant (Figure 1C). However, in *bb8-GLUD2; bb8* ^*ms*^, *bb8-GLUD1; bb8* ^*ms*^, and *bb8-Gdh; bb8* ^*ms*^ mutants, the glutamate levels are still significantly higher than those in the wild-type. The observed morphological abnormalities could explain the reduced number of offspring (Figure 2A). We also conducted paracrystalline material stability tests and found that the SDS-resistant fraction is restored in all of the transgenic constructs (Figure 1B). The observed phenotypes are reflected in transmission electron micrographs as well, and we observed mitochondrial swelling and paracrystalline material accumulation in both mitochondrial derivatives in the case of every transgenic line except *bb8* ^*GRLS*^; *bb8* ^*ms*^; however, the phenotype is not as severe as in the *bb8* ^*ms*^ mutant, and almost wild-type cysts are present (Figures 4A–C, E–I). The utilization of *bb8-GLUD2* constructs in the rescue experiment resulted in an increased progeny count, reduced mitochondrial abnormalities, and fewer abnormal migrating ICs compared to *bb8-GLUD1* and bb8-Gdh constructs (Figure 2A, Supplementary Figure S7E–G, E′–G′, Supplementary Figure S9E–G, E′–G′).

To address why the *bb8-GLUD2* construct outperformed the other transgenic constructs, we searched for similarities that only GLUD2 and Bb8 share but not Gdh or GLUD1 in the sequence alignments of the four glutamate dehydrogenases (GLUD1, GLUD2, Gdh, and Bb8) (Supplementary Figure S2). We found one amino acid (glycine-G) in position 304 (alignment), which is the same in both Gdh and GLUD1, but different in Bb8 and GLUD2 (arginine-R and lysine-K, respectively). We found three amino acids that are identical in Bb8 and GLUD2 but different in Gdh and GLUD1 in alignment position 231 (GLUD1-serine-S, GLUD2-asparagine-N, Gdh-glycine-G, and Bb8-asparagine-N), 389(GLUD1-Serine-S, GLUD2-threonine-T, Gdh-valine-V, and Bb8-threonine-T), and 528 (GLUD1-glycine-G, GLUD2-alanine-A, Gdh-glycine-G, and Bb8-alanine-A). The 231 and 389 positions are part of the conserved NADH-binding pocket, while the 528 aa position is located in the pivot helix region. We know that the S174 > N (231 in alignment) mutation in GLUD2 increased the basal activity of the enzyme and decreased the sensitivity to estrogens and neuroleptics, while the S331 > T (389 in alignment) mutation has no obvious effect on enzyme activity (Plaitakis et al., 2011; Shashidharan and Plaitakis, 2014). In position 501 (528 in alignment), the effect of alanine (A) is known for decreasing the GTP inhibitor effect as it inhibits the effect of GTP binding on allosteric regulation; therefore, it plays a role in the GTP tolerance of GLUD2 (Zaganas and Plaitakis, 2002).

Using FireProt-ASR and Bb8-like protein sequences, we estimated the ancestral sequence of Bb8 protein and investigated the amino acid changes (Musil et al., 2021). We focused our attention on changes in the previously described binding pockets. The results are summarized in Supplementary Table S2. As mentioned previously, the NADH-binding pocket has a higher similarity between Bb8 and GLUD2 proteins, and this is mainly because the GLUD2 227th and 384th amino acids are identical to the Bb8 counterparts. The predicted ancestral sequence of Bb8 protein in these positions is unchanged, suggesting that these alterations occurred early in the evolution of the protein. In GLUD2, these changes also occurred relatively early after gene duplication (S227N 18-23 Mya and S384T 14-18 Mya) (Shashidharan and Plaitakis, 2014). We also investigated the conservation of these sites using ConSurf (Supplementary Table S2B, Supplementary Table S3) (Landau et al., 2005). Meanwhile, position Bb8 206 (GLUD 227) seems to be conserved, which suggests an adaptive function, and the Bb8 363rd position is the second least conserved NADH pocket site, which suggests that the similarity might be a coincidence in its case.

We also investigated an additional site in the pivot helix in position 486 of Bb8 protein. In GLUD2, this site (509) is known for GTP regulatory inhibition (Kanavouras et al., 2007). Based on the ancestral sequence prediction, this mutation first appeared in the Drosophilidae branch. Many other GTP-binding pocket amino acid differences in Bb8 compared to those in GLUDS and Gdh (Bb8 245, 248, 293, 294, 297, 298, 301, 325, 328, 480, and 484) are also ancestral or variable. The ConSurf conservation analyses also showed only an intermediate conservation of these sites in Bb8-like GDHs; meanwhile, in general, these sites are conserved (Supplementary Table S2). Based on this, we can hypothesize that GTP inhibition was questionable in the ancient form of the Bb8 protein, but it was not because of the mutation in the pivot helix. Nevertheless, the G>A change in the pivot helix (Bb8 486) might strengthen resilience to GTP inhibition and provides a good target for theory testing.

Based on this knowledge, we decided to test the importance and effects of changes in amino acids in positions 231, 389, and 528 (Supplementary Table S2 alignment) on *Drosophila* Gdh. The *bb8*-*Gdh* construct has the weakest rescue capacity of the *bb8* ^*ms*^ phenotype. To investigate the functional similarity between bb8 and GLUD2, we changed the Gdh amino acids G501 > A (528 in alignment), G218 > N (231 in alignment), and V376 > T (389 in alignment)) to Bb8/GLUD2-like amino acids (details on Figure 1A) and tested the rescue capacity of the transgenes (Figure 1A). All of the new Gdh mutants showed partial rescue of the mutant *bb8* ^*ms*^ phenotype and produced significantly more offspring than the wild-type *bb8*-*Gdh* flies (Figures 1B,C; Figure 2B; Figure 3, Supplementary Figure S5–S9A, B, G–J, A′, B′, G′–J′). We found that the G501 > A transition showed the weakest rescue capacity out of the three Gdh mutants, and the triple-point mutant has improved fertility compared to G501 > A. Interestingly, the alteration of the NADH-binding pocket without the G501 > A mutation showed the best rescue (Figure 2B). We believe that the method used in this study to measure glutamate levels might not be sensitive enough to precisely show the differences between the Gdh variants; however, their effect on the offspring number and spermatid development is significant.

To further analyze the G218 > N and V376 > T mutations and their possible role in the enzyme function, we gathered experimental and *in silico* structural data on glutamate dehydrogenases. Structural studies showed that glutamate dehydrogenases are flexible molecules as they can have opened and closed conformations (Supplementary Figure S10, 3jd3 and 3jd4 models) (Borgnia et al., 2016). The open and closed conformations can switch depending on the allosteric regulatory factors (ADP and GTP) and other substrates bound by the enzyme (Borgnia et al., 2016). It is assumed that the active site of the open form is more accessible to substrates due to the wider spaces present, whereas the closed conformation holds the substrate in place, and the reaction can proceed in this form. The subsequent conformational change releases the enzyme product (Supplementary Vidoe S1). *D. melanogaster* Gdh has no available crystal structure, but AlphaFold software predicted the Gdh and Bb8 structures with high confidence. Comparing these models to the opened and closed conformations of the bovine glutamate dehydrogenase models, we find that they have an intermediate conformation, which is most probably because the prediction does not take co-factors and their effect on the conformation into account. However, to perform a comparative analysis, it would be beneficial to have molecule models in the same conformation; therefore, we created further models using MODELLER. The Gdh structure generated that way is very similar to the template molecule (6DHN), differing significantly only in the antenna region (Supplementary Figure S10) (Bailey et al., 2011).

**FIGURE 5:**
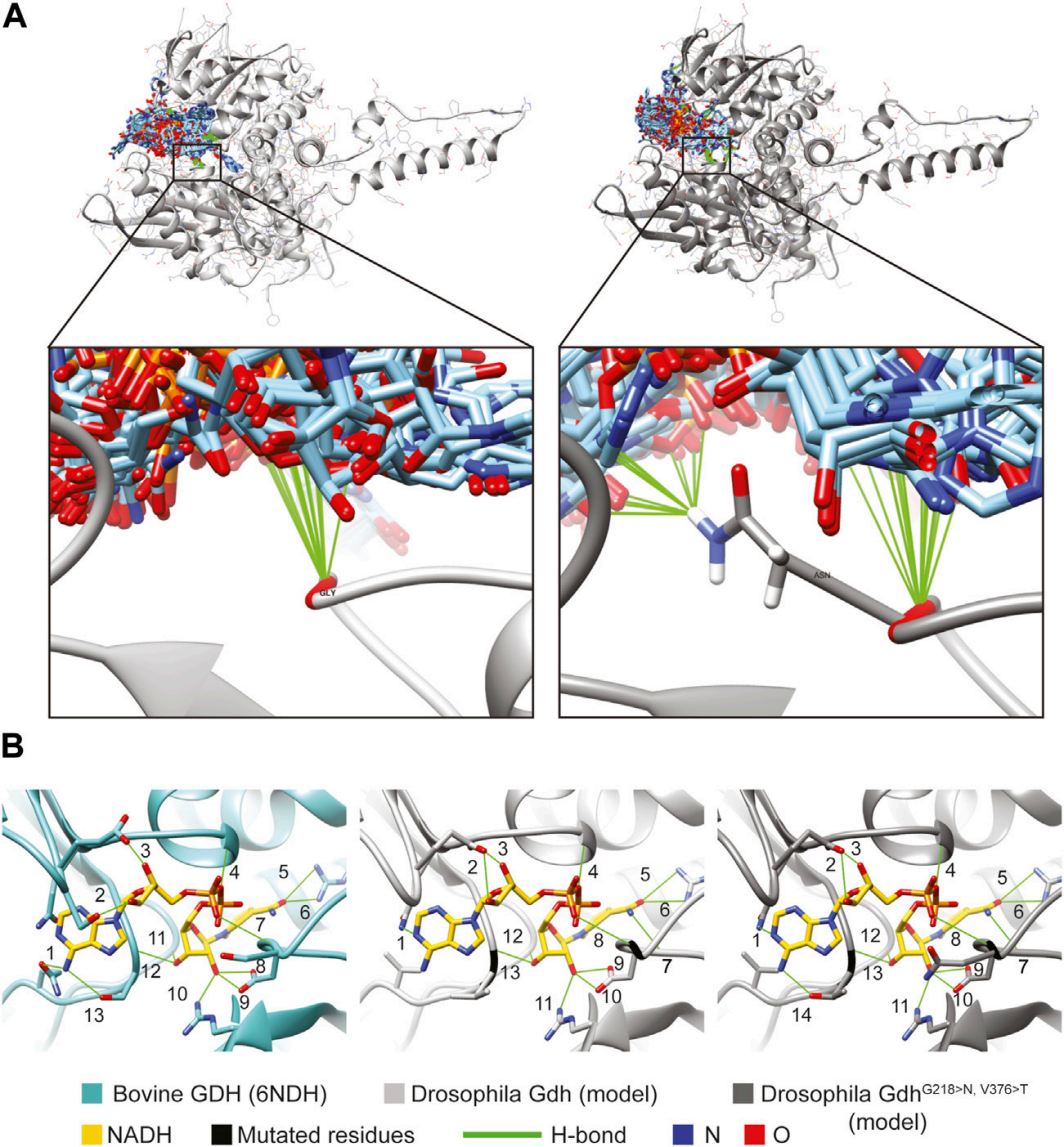
Molecule model of Gdh and NADH positions. **(A)** SwissDock results show possible positions of 276 NADH molecules (light blue) to the *Drosophila melanogaster* Gdh model. Both Gly of the wild-type (light gray) and Asn of the mutant version (dark gray) of fly Gdh are found to form H-bonds in several cases. **(B)** NADH is positioned by 13 H-bonds in the active centrum of bovine GDH (6NDH). Similarly, we can find several H-bonds with the overlapped Gdh models of *Drosophila*: 13 in the wild-type and 14 in the mutant.

We used this model to predict NADH binding in the wild-type and mutant Gdh using SwissDock (Grosdidier et al., 2011). The NAD positions did not overlap with that of the template model. However, the glycine (G wild-type) and the asparagine (N mutant) amino groups form H-bond in several models. Asparagine, due to its side chain, could form further H-bonds, suggesting that it could play a role in keeping NAD in the channel to the active centrum or guiding it to the correct position (Figure 5, Supplementary Table S1C,D).

The *Drosophila* Gdh model in superposition with the bovine GDH crystal structure complexed with NADH allowed us to examine the possibility of H-bond formation and clashes between the Gdh (wild-type and mutant) models and NADH from the template molecule. There were no clashes detected; however, several H-bonds were found and could stabilize NADH in the active centrum. In the template (6DHN), we found 13 H-bonds, and it was the same in the case of wild-type Gdh. The valine–threonine (V376 > T) mutation creates the possibility to form a further H-bond, suggesting that mutant Gdh might have a higher affinity to NADH (Figure 5B #14). In the comparative model, glycine and asparagine both form H-bonds similar to SwissDock models (Figure 5 H-bonds marked with #7 and 8). The presented models might serve as an explanation of why these mutations are beneficial; however, further experiments are needed to validate these results.

### The role of glutamate during post-meiotic development

**FIGURE 6:**
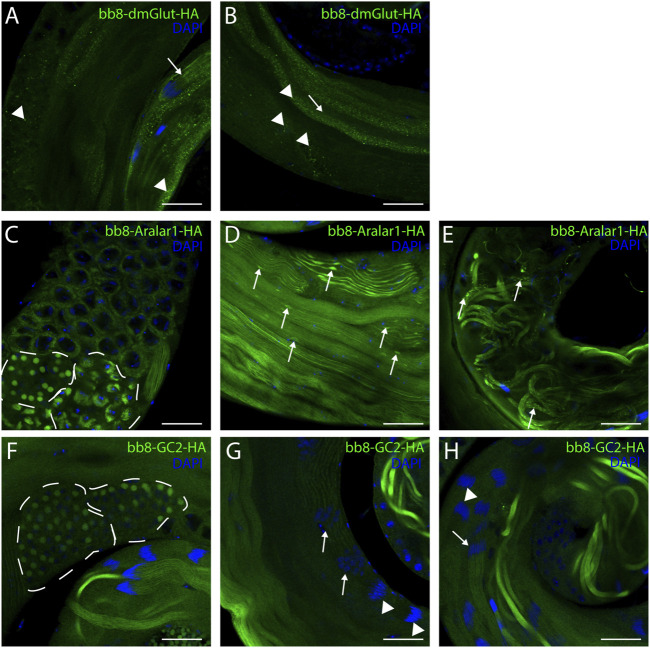
Confocal microscopic images of intact testes expressing transgenic constructs. Green represents Bb8-driven transgenic constructs (dmGlut-HA A, B, Aralar1-HA C-E, and GC2-HA F-H) visualized by anti-HA immunostaining. DAPI staining highlights the nuclei. **(A, B)** Arrows highlight membrane-bound localization patterns, and arrowheads point to aggregates. **(D, E)** Arrows represent mitochondrial abnormalities. **(C, F)** Dashed lines highlight meiotic cysts and cysts containing early-round spermatids. **(G, H)** Arrows point to cysts which show staining, and arrowheads point to cysts where no signal was observed. Scale bars represent 40 μm.

Next, we raised the question of whether the elevated glutamate level is sufficient for megamitochondria formation in spermatids. To create elevated glutamate levels in spermatids or in mitochondrial derivatives independently from Bb8 glutamate dehydrogenase, we turn our attention to *Aralar1*, *glutamate carrier 2* (*GC2*), and *dietary and metabolic glutamate transporter* (*dmGlut*). Both *Aralar1* and *dmGlut* have a minimal expression in the testis (Shim et al., 2011; Leader et al., 2018; Vedelek et al., 2018; Lunetti et al., 2021). dmGlut is a transporter located in the plasma membrane, and its overexpression in S2 cells can cause elevated glutamate levels and megamitochondria formation (Shim et al., 2011). Aralar1 is a mitochondrial glutamate transporter that exchanges the mitochondrial aspartate for cytosolic glutamate in a calcium-dependent manner (Lunetti et al., 2021), while GC2 is predicted to be a testis-specific mitochondrial glutamate transporter (Lunetti et al., 2013). By the overexpression of dmGlut, we would expect elevated glutamate levels inside the cysts, which could affect mitochondrial morphology. In the case of GC2 and Aralar1, we expect elevated glutamate levels inside the mitochondria. To test the effect of Aralar1, dmGlut, and GC2 in the stages where Bb8 is active, they were expressed with the *bb8* regulatory regions and with a single C-terminal HA-tag, which helped visualize the expression pattern of the transgenes (Figure 1A; Figure 6). We expect the transgenes to be expressed in the same developmental stages; therefore, the observed differences might be the consequence of the different localization and stability of these proteins. The ectopically expressed Aralar1-HA is present in mature spermatocytes, and round and elongating spermatids, while GC2-HA showed a weak expression pattern in the round spermatids and a stronger accumulation in the already elongated spermatids. dmGlut-HA showed a weak signal, mostly observable on the spermatid membrane, and in many cases it formed aggregates. We found that the ectopically expressed dmGlut-HA and GC2-HA have negligible influence on fertility and elongation (Figure 2C, Supplementary Figure S8, A, K, L, N). However, we found some minor mitochondrial abnormalities in the spermatids of bb8-GC2-HA flies (Supplementary Figure S5–S7A, K, L, A′, K′, L′) and a minor disturbance in individualization (Figure 3, Supplementary Figure S9K, L, K′, L′, N). Overall, we believe the dmGlut-HA and the GC2-HA constructs’ influence on spermatogenesis is questionable; the observed aggregates in dmGlut might indicate structural problems of the transgene. Meanwhile, we know little about GC2 functionality. In contrast to *bb8-dmGlut-HA* and *bb8-GC2-HA*, the ectopic expression of *bb8-Aralar1-HA* causes a significant decrease in the offspring number. We observed megamitochondria formation, a disturbance in elongation, and individualization in the transgenic spermatids (Figure 2C; Figure 3, Supplementary Figure S5–S9M, M′). The observed phenotype is very similar to the *bb8* ^*ms*^ mutants. Based on literature data and the presented observations, this phenotype can be explained with the disturbance of glutamate homeostasis. A fluorimetric measurement of glutamate showed a significant reduction in the total amount of glutamate in *bb8-Aralar1-HA*-expressing testes compared to the amount in wild-type (Figure 1C). This further proves the functionality of the *bb8-Aralar1-HA* transgene as the Aralar1 protein imports the cytosolic glutamate to the mitochondria, where its local accumulation can cause mitochondrial abnormalities. However, the Bb8 glutamate dehydrogenase activity is unaffected in these mutants, resulting in the catabolism of the glutamate transported to the mitochondria. It is plausible that Aralar1 imports glutamate at a higher rate than Bb8 can catabolize it. Therefore, we can observe the mitochondrial abnormalities caused by elevated glutamate presence in mitochondria; meanwhile, the overall glutamate pool decreases due to mitochondrial glutamate dehydrogenase activity.

## Discussion

**FIGURE 7:**
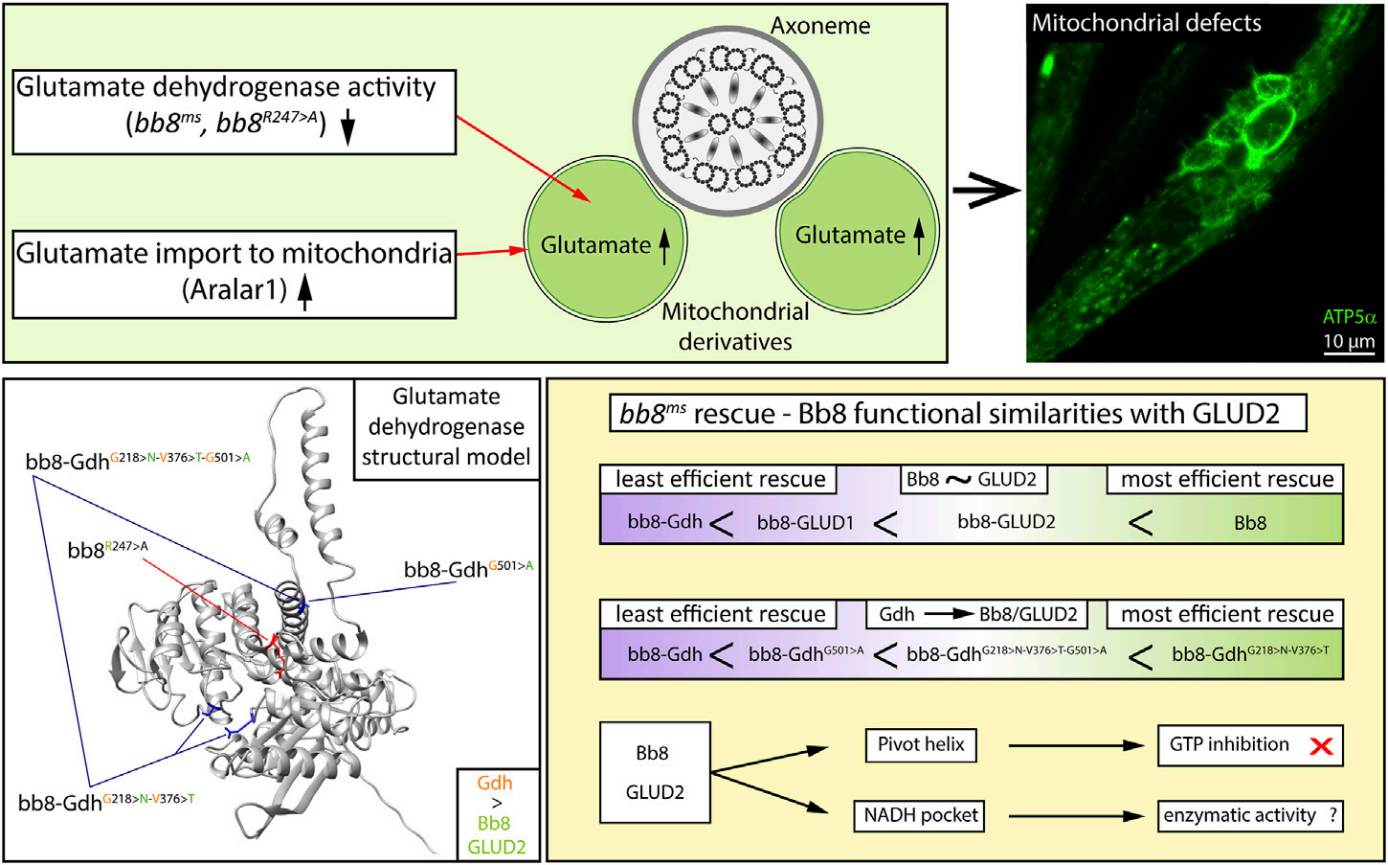
Graphical summary.

Gene duplication opens the way for the neofunctionalization of proteins, which can drive evolution (Roth et al., 2007; Wagner, 2008). In multiple cases, it was shown that newly emerged genes or gene duplications gain testis-specific functions in *Drosophila* (Kondo et al., 2017). Based on the phylogenetic tree of glutamate dehydrogenases, we can see that the Bb8-like genes of Drosophilidae are on a distant branch, while mammalian gene duplicates are much younger and closer to the tree (Supplementary Figure S3). Vertebrae duplicates are generally closer to each other; however, we can observe an additional, more distant branch of glutamate dehydrogenases (including reptile and bird glutamate dehydrogenases); for example, the glutamate dehydrogenases of *Haliaeetus leucocephalus* are on two distant branches. We find this similar to what we observed in the case of Bb8-like glutamate dehydrogenases. Overall, we can hypothesize that the emergence of more than one glutamate dehydrogenase in a single organism happened multiple times, and Bb8 and Bb8-like proteins are a variation of such an event, which is present in fly and mosquito species. The emergence of gene duplicates opens the possibility for the duplicate to gain new functions, like in the case of human GLUD1 and GLUD2. S-Lap proteins are a good example for neofunctionalization in *Drosophila*, where the paralogous S-Lap genes lost their enzymatic function and became structural elements of the paracrystalline material of the major mitochondrial derivative. The paracrystalline material also contains the Bb8 protein. In contrast to the S-Lap proteins, we showed that Bb8 kept its enzymatic activity. Bb8 enzymatic activity is functionally different from the housekeeping Gdh activity as the *bb8*-*Gdh* transgenic rescue was not able to sufficiently restore wild-type fertility. Based on *in silico* modeling, there is a structural similarity between Bb8 and other glutamate dehydrogenases. The enzymatic active centrum of Bb8 consists of conserved amino acids, and experimental evidence showed elevated glutamate levels in *bb8* ^*ms*^ testes. Interestingly the *bb8* ^*R247>A*^ mutation in the active centrum of the enzyme was capable of the partial rescue of the *bb8* ^*ms*^ mutant phenotype. Despite the hypomorphic nature of the allele, mitochondrial abnormalities are present in the *bb8* ^*R247>A*^; *bb8* ^*ms*^ mutants. As we reported previously, in *bb8* ^*ms*^ mutants, the mitochondrial derivatives do not differentiate properly, and both start to accumulate paracrystalline material (Vedelek et al., 2016). The electron micrographs of *bb8* ^*R247>A*^; *bb8* ^*ms*^ mutants revealed paracrystalline material accumulation defects, which suggests an important role for the enzymatic activity of Bb8 in the structural development of mitochondrial derivatives. Based on this, we presume that the differentiation of mitochondrial derivatives also needs specific metabolite content; therefore, the mitochondrial metabolite composition may play a role in proper paracrystalline material formation. It seems that the disturbance in glutamate homeostasis easily leads to abnormal mitochondrial morphogenesis either due to the lack of Bb8 or the ectopic expression of the mitochondrial carrier Aralar1. As we reported previously, in *bb8* ^*ms*^ mutants, the mitochondrial derivatives do not differentiate into major or minor derivatives, and we observed similar defects in *bb8* ^*R247>A*^ *; bb8* ^*ms*^ mutants. We conclude that the role of Bb8 in the spermatids is to catabolize glutamate to alpha-ketoglutarate, most likely providing additional material to the TCA cycle, and by regulation of metabolites, it establishes a niche for proper paracrystalline material formation (Figure 7).

We provide experimental evidence that the Gdh enzyme is not able to rescue the *bb8* ^*ms*^ mutant; therefore, we concluded that there is a functional difference between the *Drosophila* housekeeping Gdh and Bb8. There is a known functional difference between GLUD1 and GLUD2; therefore, we can state that both tissue-specific *Drosophila* and human GDHs have diverged from their housekeeping counterparts. The evolutionary distant human glutamate dehydrogenases, the housekeeping GLUD1, and the tissue-specific GLUD2 showed a more efficient rescue capacity than Gdh based on fertility and phenotypical analyses (mitochondrial abnormalities and individualization defects). In enzymatic pathways, allosteric regulation is key for the tuning of enzymatic activity and maintaining metabolic homeostasis. In the case of glutamate dehydrogenases, a wide variety of allosteric regulations are known (Smith and Stanley, 2008; Li et al., 2012). The human GLUD1 and GLUD2 enzymes have different regulations despite the very high sequence similarity, which can also explain why bb8-GLUD2 shows a better rescue than bb8-GLUD1. In contrast to this, Gdh and Bb8 have relatively low sequence similarity, which makes us assume that they are more differentiated enzymes. The superior rescue capacity of the bb8-GLUD2 construct over bb8-GLUD1 and bb8-Gdh suggests that there is a functional similarity between the two tissue-specific glutamate dehydrogenases Bb8 and GLUD2, which might be a result of convergent evolution, and they might function in similar cellular environments with similar molecular regulatory mechanisms. If these enzymes occupy a similar molecular niche, they adapt in response to similar selective pressures, which can explain their functional similarity. The G528 > A amino acid transition in the pivot helix of GLUD2 is known to be required to avoid GTP inhibition (Zaganas and Plaitakis, 2002). Similarly, Bb8 also contains the A528 amino acid in the pivot helix; furthermore, the Bb8 GTP-binding pocket is the least conserved compared to Gdh, GLUD1, and GLUD2. Furthermore, Bb8-like proteins have less conserved GTP pockets compared to 150 orthologous GDH sequences. By introducing the G501 > A (528 in alignment) mutation in the pivot helix of bb8-Gdh^G501>A^, we significantly increased the number of offspring compared to that of bb8-Gdh. This line of evidence strongly suggests that similar to GLUD2, Bb8 has limited GTP sensitivity, and we can suppose that during spermatid differentiation, there might be glutamate catabolism even in the presence of GTP. It is likely that both GLUD2 and Bb8 cast off the shackles of GTP regulation; however, for further clarity, biochemical analysis of purified Bb8 enzyme activity would be necessary (Figure 7).

Additionally, the better rescue capacity of bb8-GLUD2 could be due to the similarity between the Bb8 and GLUD2 sequences in the NAD(H) pockets N231 and T389. Interestingly, the changes in the NAD(H) pocket greatly increased the rescue capacity of the *bb8-Gdh* ^*G218>N−V376>T*^ transgene. It is known that the GLUD2 S174 > N mutation increases the basal activity of the enzyme (Plaitakis et al., 2011). We believe the altered amino acids either facilitate the exchange of the substrates or increase the efficacy of enzymatic reaction by altering the enzyme affinity to NADH; however, the precise mechanism is not yet studied in detail.

In the bb8-Gdh^G218>N−V376>T−G501>A^ triple mutant, one would expect a synergistic effect of the amino acid changes; however, the G501 > A transition seems to inhibit the effect of the G218 > N and V376 > T transitions. This suggests that there could be a cost–benefit ratio with the individual mutations, and while individually they increase the efficacy of rescue, in combination, they are less effective. The G501 > A transition has a strong impact on the conformation of open and closed forms of the GLUD enzymes; therefore, its alteration in Gdh might affect the NAD-binding pocket, which causes less-efficient rescue (Zaganas and Plaitakis, 2002). Alternatively, these mutations might influence an additional function next to enzymatic activity, which is paracrystalline material formation. It is important to note that it is challenging to distinguish between the structural and enzymatic roles since megamitochondria formation and paracrystalline material accumulation happen simultaneously in the elongating spermatids (Figure 7).

There are multiple pieces of evidence that GLUD2 is important in brain development. GLUD2 expression is higher in human newborns compared to that in chimpanzee newborns, and the glutamate content is lower than in chimpanzees and macaques (Fu et al., 2011). The transgenic expression of GLUD2 influences the early post-natal development of mice brains (Li et al., 2016). The role of GLUD2 is debated in brain development; on one hand, GLUD2 might play a role in synaptogenesis due to its capability to support intense glutamatergic activity and, therefore, play a role in brain plasticity (Plaitakis et al., 2019). On the other hand, alpha-ketoglutarate provided by glutamate catabolism can facilitate the biosynthesis of lipids because excess acetyl-coenzymeA could be utilized in the lipid biosynthesis pathway (Li et al., 2016; Plaitakis et al., 2019; Yoo et al., 2020). This is beneficial for growth and the lipid supply of the developing brain. In *Drosophila* testes, the metabolic role seems to be a viable option for glutamate dehydrogenase activity as the extreme length of sperm required excessive amounts of lipids (Tokuyasu, 1974; Laurinyecz et al., 2016). However, it is an open question whether the elongation defect we observed in *bb8* ^*ms*^ mutants is a consequence of metabolic issues or morphological abnormalities.

The sodium salt of glutamate is monosodium glutamate (MSG), which is responsible for the umami taste and is used as a major flavor enhancer. It is generally regarded as safe for human consumption; however, MSG poisoning might occur in susceptible individuals (allergy) and cause a variety of symptoms like headache, flushing, or chest pain (Kazmi et al., 2017). More interestingly, MSG can cause male reproductive toxicity in rodents with a wide variety of symptoms (Kayode et al., 2020). MSG’s effect on human reproduction is unknown (Kayode et al., 2020). Nevertheless, studies in rodents raise the possibility that elevated glutamate levels have a generally negative effect on spermatogenesis and that glutamate metabolism is crucial for proper spermatogenesis, not only in *Drosophila* but likely in mammals as well.

## Materials and methods

### Fly maintenance

*Drosophila* stocks were maintained on standard cornmeal media at 25°C. We used the following fly stocks from the Bloomington *Drosophila* Stock Center: Mi{ET1}bb8^MB10362^ (BDSC 27841), W1118, y1 M{nos-Cas9.P}ZH-2Aw- (BDSC 54591), and y^1^ v^1^ P{y^+t7.7^ = nos-phiC31\int.NLS}X; P{y^+t7.7^ = CaryP}attP40 (BDSC 25709). The remaining stocks were established for this study.

### Fertility tests

For fertility assays, individual males were crossed with five or three (indicated in figure legends) wild-type virgin females. After 5 days, the parents were removed from the vials. Offspring were counted on the day 12 after crossing. Boxplots represent multiple experiments, where each experiment’s offspring count was min-max normalized between 0 and 100. Welsch’s two-tailed significance test was used to determine significance. Plot and related statistical analysis were created using Python 3.0 with NumPy, pandas, Seaborn, and SciPy libraries.

### Molecular and biochemical methods

Mitochondrial signal peptides were determined using TargetP 2.0 and MitoFates (Fukasawa et al., 2015; Almagro Armenteros et al., 2019). The oligonucleotide primers used for this study are listed in Supplementary Table S4. We used the *w* ^*1118*^ DNA extract to amplify *Drosophila bb8* (and regulatory regions), *GC2*, and *w* ^*1118*^ cDNA for *Aralar1*, *dmGlut*, and *Gdh* genes. We used HeLa cell DNA and RNA extracts to amplify GLUD2 and GLUD1, respectively. Phusion high-fidelity DNA polymerase (New England BioLabs) was used for PCR reactions. Purified PCR products were cloned into the pUASTattB vector using the HiFi DNA Assembly Master Mix (E2621S, NEB) (Gibson et al., 2009). Point mutations were introduced by PCR mutagenesis, and purified PCR products were self-ligated with T4 ligase (Thermo Fisher Scientific) and cloned. All transgenic constructs were inserted to the attP40 landing site to *y, w* ^*1118*^ *; P{CaryP}attP40* (BDSC 25709) flies using a standard germline transformation technique. All constructs were Sanger-sequenced to check proper sequences.

Polyacrylamide gel electrophoresis and the paracrystalline material stability test were performed, as described by Laurinyecz et al. (2019b). Samples were loaded on 10% polyacrylamide gel and stained with Coomassie brilliant blue (Thermo Fisher Scientific).

Glutamate levels were measured using an Amplex Red glutamic acid/glutamate oxidase assay kit (Thermo Fisher Scientific), according to the manufacturer’s instructions. For each sample, 10 pairs of testes were dissected from 1–2-day-old males, and samples were stored in liquid nitrogen. Testes were homogenized in 200 μL 0.5M Tris-HCl, pH 7.5, by sonication. Sonics Vibra-cell CV18 was used with a 3-mm probe at 40% amplitude for 2 × 20 s with a 20-s gap between. If the sample was not fully homogenized, we treated the sample for an additional 20 s. Each genotype was represented with at least four biological samples, and each sample was tested in duplicates. In 96-well plates, 50 μL of samples were used in each reaction, which were incubated at 37°C for 15 min. Fluorescence was measured on a Synergy HTX multi plate reader. Plate reader results were processed based on 0 μM, 5μM, 10 μM, 15 μM, and 20 μM calibration curves, and concentrations were estimated based on trend lines using MS Excel 2016. Concentrations were normalized to one pair of testis. Graph and statistical analysis were created by Python 3.0 with NumPy, pandas, Seaborn, and SciPy libraries using Welcsh’s two-tailed significance test.

### Phylogeny, ancestral sequence prediction, and determination of conserved sites

Metazoa GDH (217293at33208) protein sequences were downloaded from OrthoDB v10.1, and species with multiple GDHs were filtered for further analysis (Kriventseva et al., 2019). Phylogenetic trees were built using the NGPhylogeny.fr website (MAFFT alignment, BMGE alignment curation, and FastME Tree inference) (Lemoine et al., 2019). The data were then exported to iTol for further visualization options (Letunic and Bork, 2021).

For SNAP analyses, we used the clustalW codon alignment (MegaX) on the CDS of 13 Bb8 and 11 Gdh–like genes from Drosophilidae (Kumar et al., 2018). For the investigation of the synonymous and nonsynonymous substitutions, we used the online SNAP v2.1.1 server (www.hiv.lanl.gov) (Korber et al., 2000).

We used FireProt-ASR to determine the sequence ancestry of Bb8, using the Bb8-like protein sequences of *Drosophila* fly and mosquito species (Musil et al., 2021). For the determination of conserved sites, ConSurf was used, and for general overview, the HMMER algorithm searched orthologs in five iterations; then, for modeling, the 150 closest hit was utilized (Landau et al., 2005; Yariv et al., 2023). For the targeted background, we used homologous sequences extracted from OrthoDB. The list of background sequence IDs is available in Supplementary Table S3.

### Comparative modeling and docking

*Drosophila melanogaster* glutamate dehydrogenase (Gdh and Bb8)-predicted structures were downloaded from the AlphaFold database (Jumper et al., 2021). InterEvDock3 was used to create homotrimer and homohexamer complexes of Gdh and Bb8(54). We used the AlphaFold 2.0 Q9VCN3 Bb8 model to highlight the conserved pockets.

All the models were compared with several bovine glutamate dehydrogenase structures (6DHN, 3JD3, and 3JD4) using UCSF Chimera (Pettersen et al., 2004).

The *D. melanogaster* Gdh amino acid sequence was obtained from FlyBase (FBpp0088990), while the corresponding *Bos taurus* sequence was extracted from the 6DHN model of glutamate dehydrogenase from PDB. The sequences were aligned in UGENE (Unipro) using the T-coffee algorithm with standard settings (Okonechnikov et al., 2012). Based on the alignment and the 6DHN structure, the *D. melanogaster* Gdh structure was predicted by MODELLER using UCSF Chimera (Sali, 1995; Pettersen et al., 2004). The best model (with the zDOPE score 1.12) was selected for further examination. Amino acids of Gdh were manually aligned with the 6DHN structure in the active centrum. The mutant versions of Gdh (Gly172Asn and Val330Thr) were also created, and the models were tested for clashes and H-bonds between the active centrum and NADH.

SwissDock was used to dock models created by a modeler with NADH *in silico* (Grosdidier et al., 2011).

### Staining and microscopy

*Drosophila* testis sample preparation and staining were performed, as described previously by White-Cooper and Henderson (2004). Phalloidin Texas Red-X (Thermo Fisher Scientific) was used at a 1:250 dilution. MitoTracker Red CMXRos (0.5 μM in PBS) (M7512, Thermo Fisher Scientific) was used on living testis samples for up to 20 min. For immunostaining, rat anti-HA (1:200) (11867423001, Roche), mouse anti-ATP5α (1:200) (15H4C4, #ab14748 Abcam), mouse anti-pan polyglycylated tubulin antibody (1:5,000) (clone AXO49, Merck), and primary, anti-rat, and anti-mouse Alexa Fluor 488 (Thermo Fisher Scientific) secondary antibodies were used at a 1:400 dilution.

Images were taken using the Olympus FLUOVIEW Fv10i confocal microscope (Olympus FW10-ASW ver. 04.02) or Olympus BX51 fluorescent microscope (Olympus cell^A ver. 3.3 software). Images were processed with GIMP 2.8.6, and the length of AXO49-positive cysts was measured using ImageJ software.

Electron microscopic analysis of testes was carried out, as described by Laurinyecz et al. (2016).

## Data Availability

The original contributions presented in the study are included in the article/Supplementary Material; further inquiries can be directed to the corresponding authors.
