# Supplementary material for: A comparative analysis of fruit fly and human glutamate dehydrogenases in Drosophila melanogaster sperm development
Source: Front Cell Dev Biol. 2023 Nov 2;11:1281487. doi: 10.3389/fcell.2023.1281487 (PMC10652781; doi:10.3389/fcell.2023.1281487)

Supplementary Figure S2

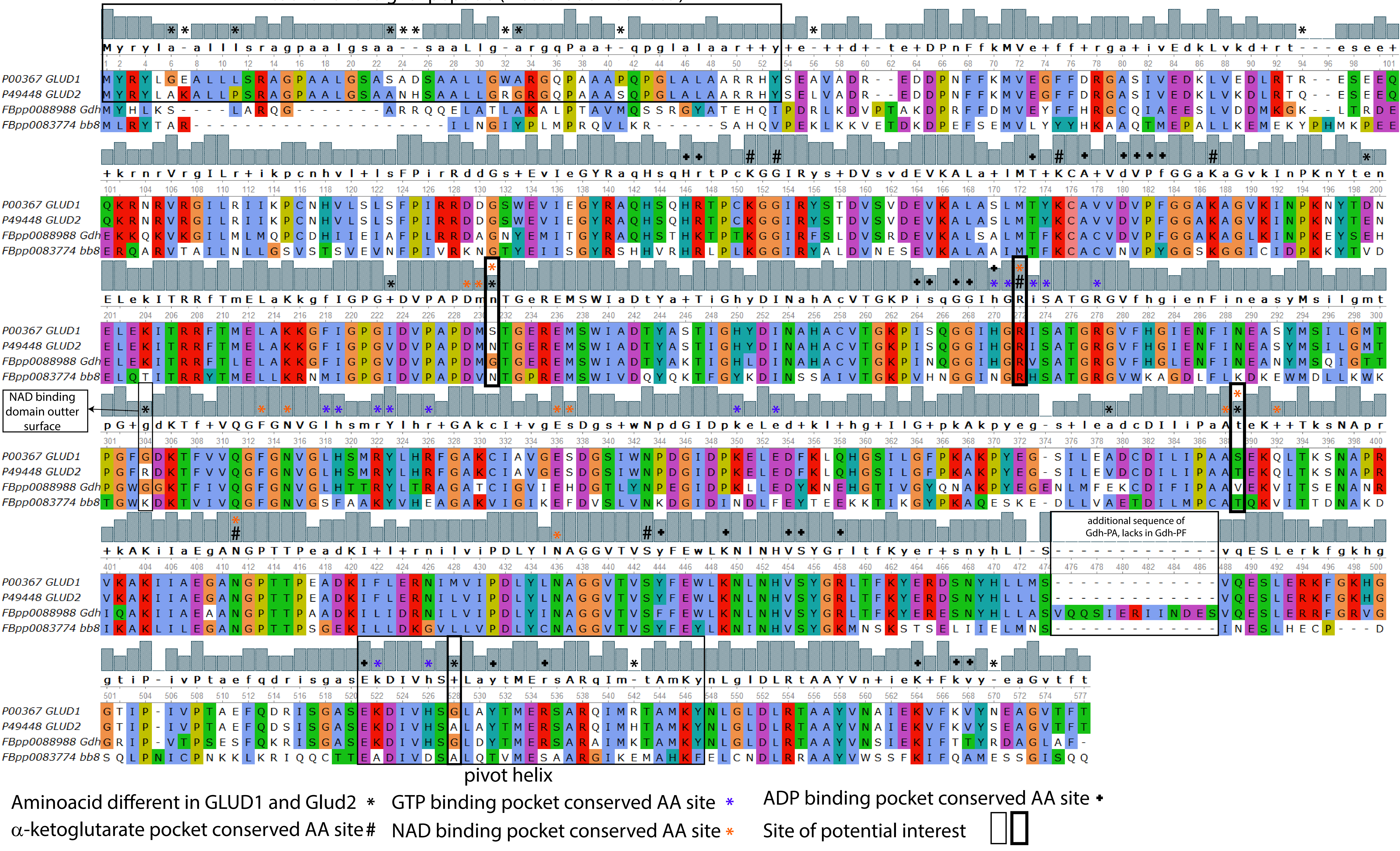

Supplement: Supplementary file 3 [file Presentation1.zip › Image 2.PDF]
