# Supplementary figures and images for: A comparative analysis of fruit fly and human glutamate dehydrogenases in Drosophila melanogaster sperm development
Source: Front Cell Dev Biol. 2023 Nov 2;11:1281487. doi: 10.3389/fcell.2023.1281487 (PMC10652781; doi:10.3389/fcell.2023.1281487)

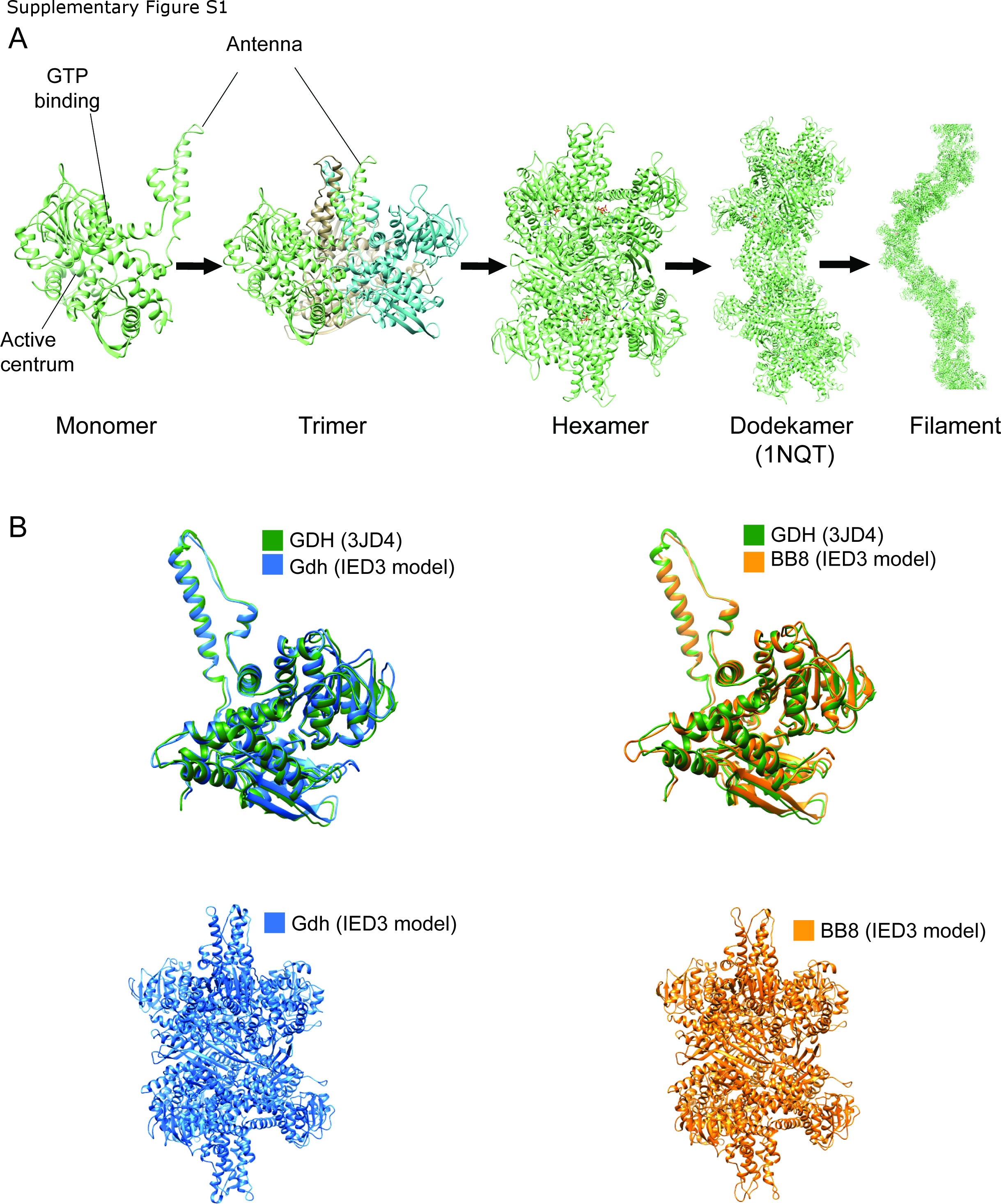

Supplement: Supplementary file 3 [file Presentation1.zip › Image 1.TIF]

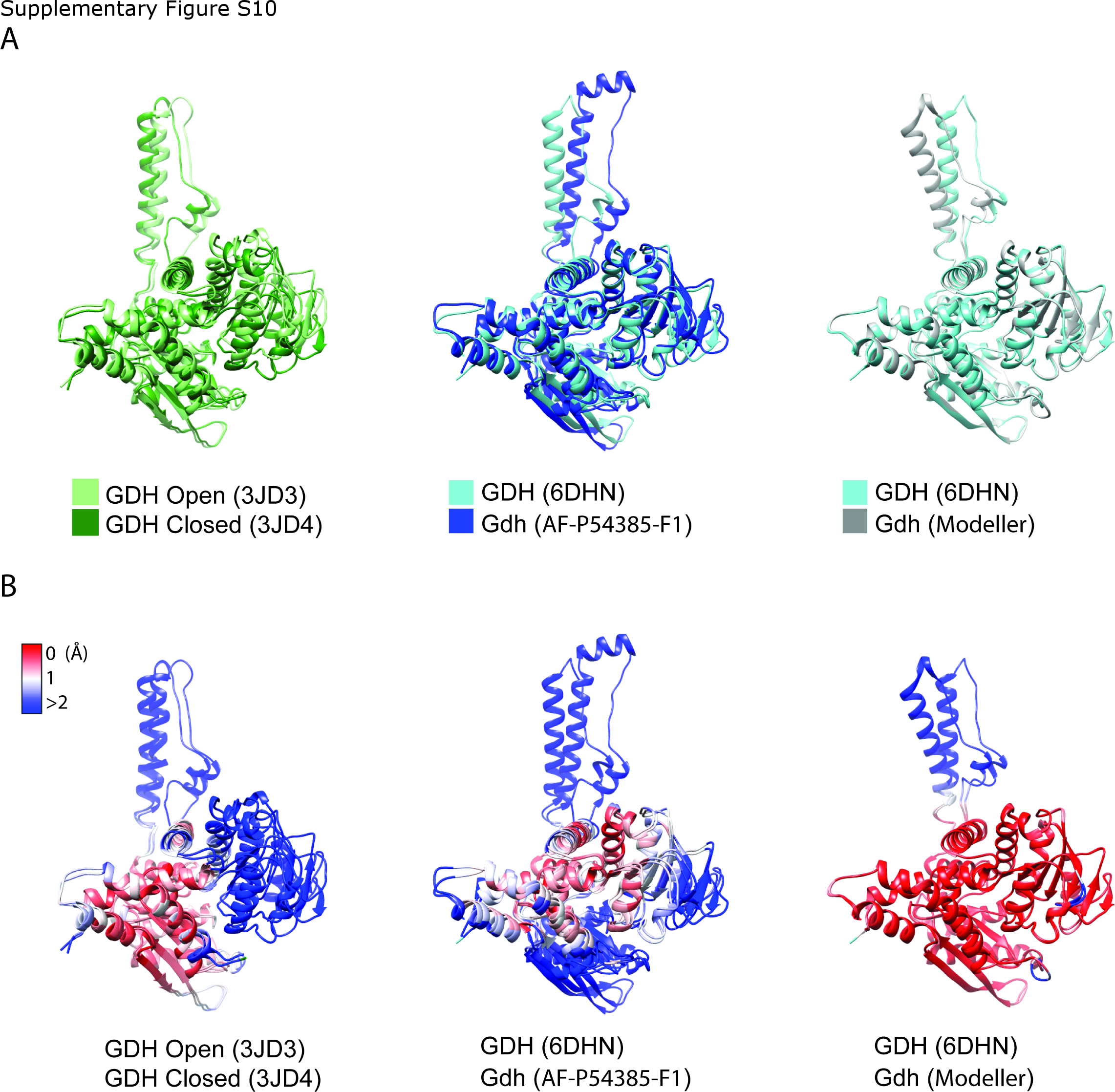

Supplement: Supplementary file 3 [file Presentation1.zip › Image 10.TIF]

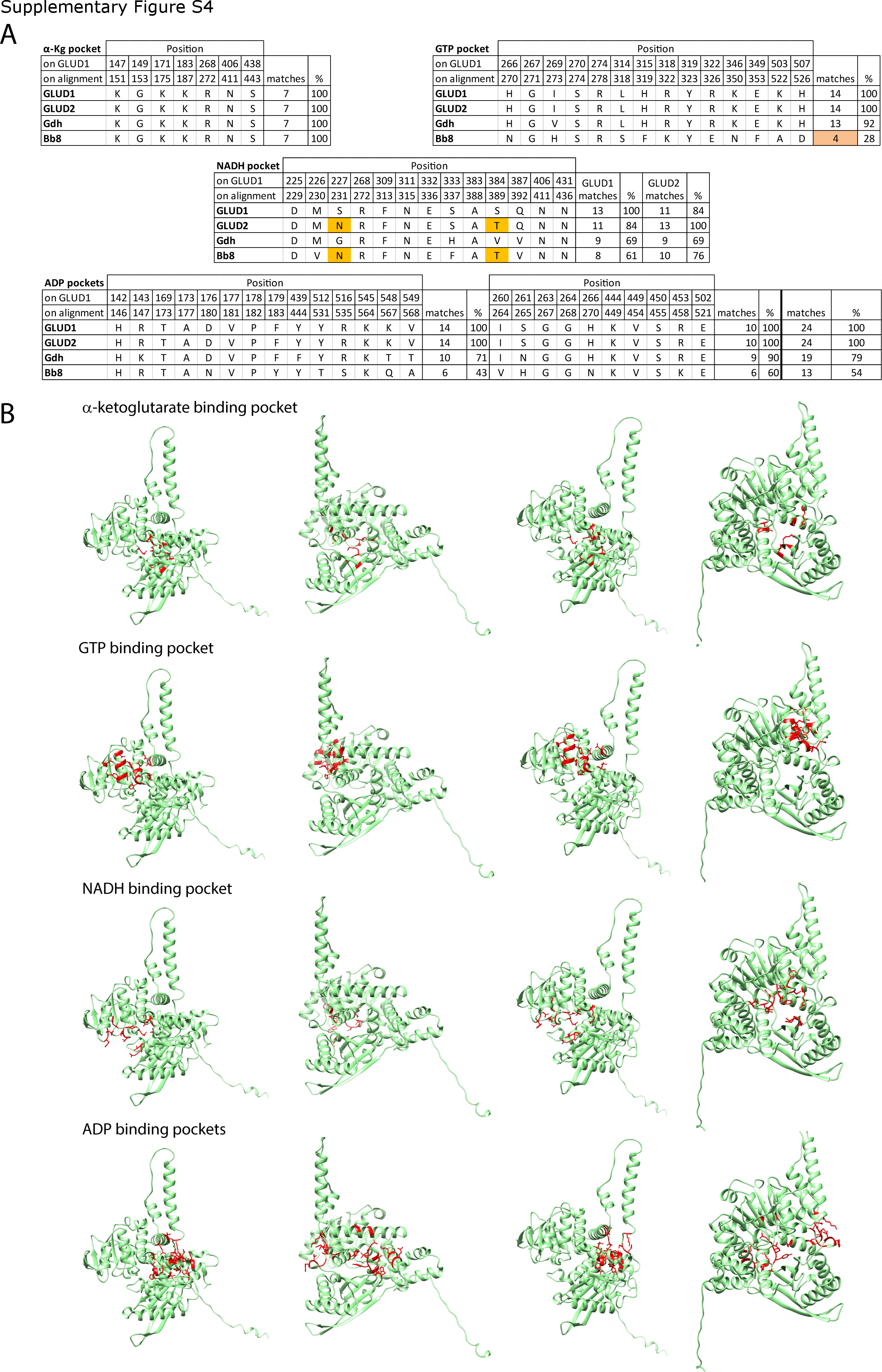

Supplement: Supplementary file 3 [file Presentation1.zip › Image 4.TIF]

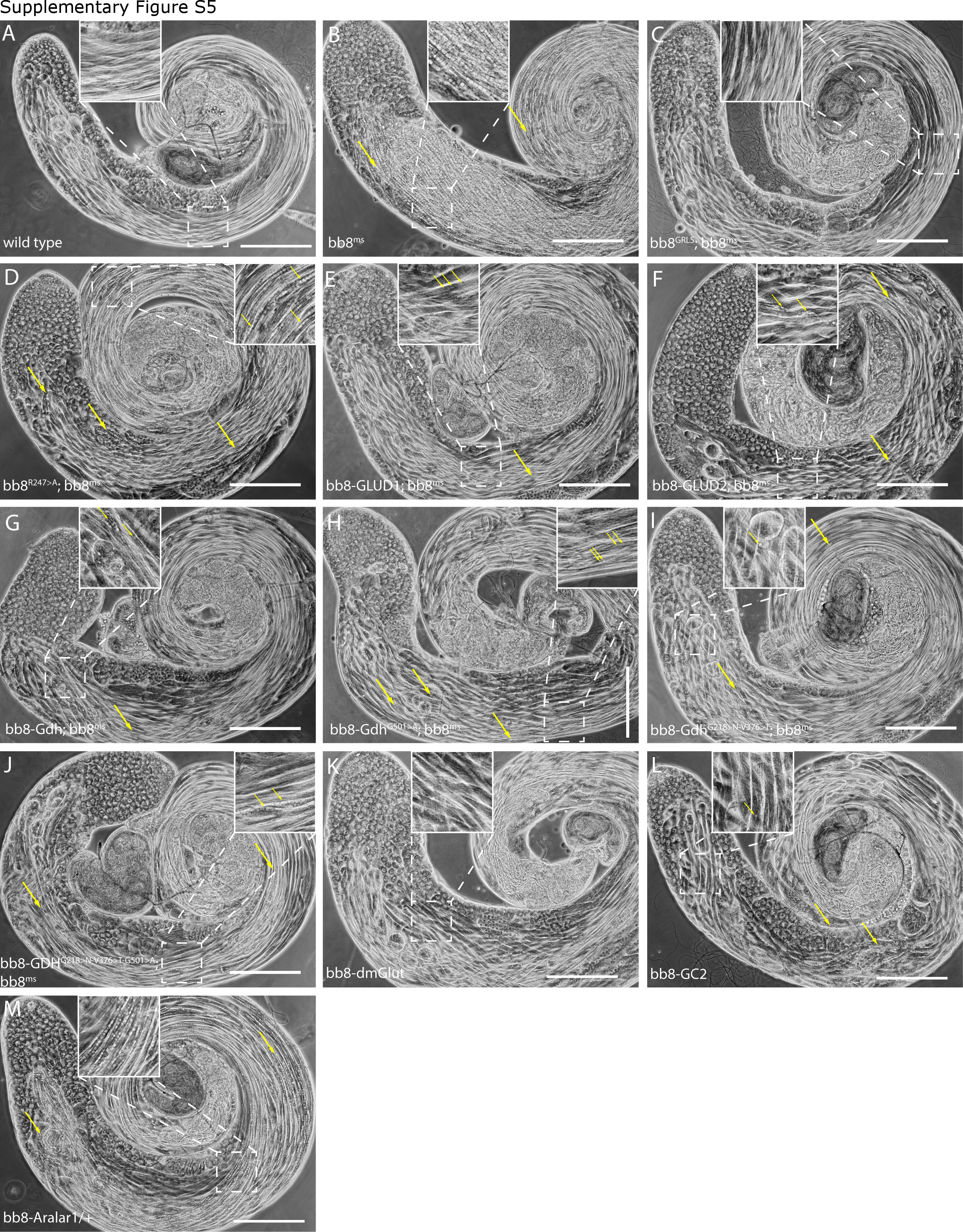

Supplement: Supplementary file 3 [file Presentation1.zip › Image 5.TIF]

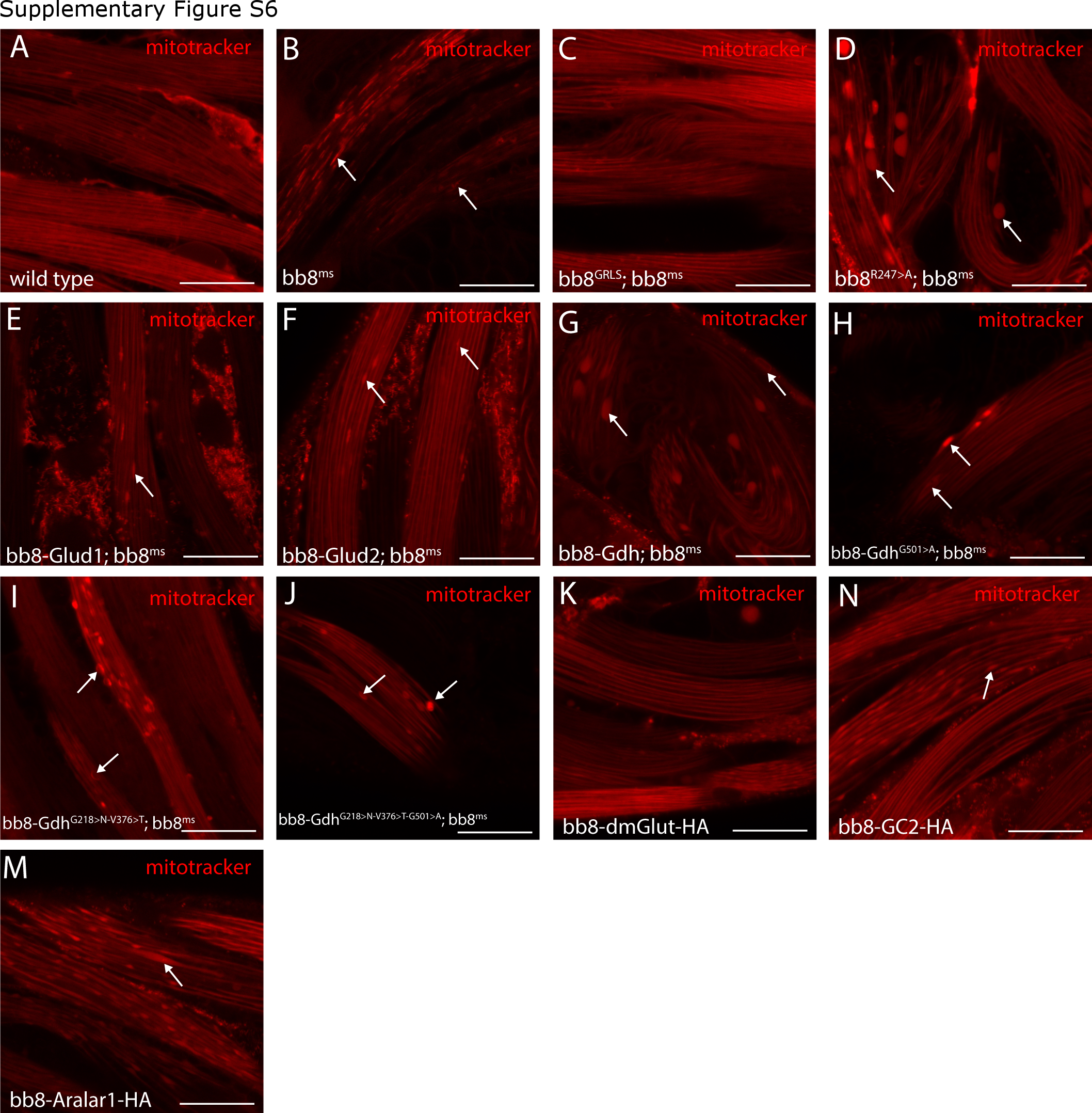

Supplement: Supplementary file 3 [file Presentation1.zip › Image 6.TIF]

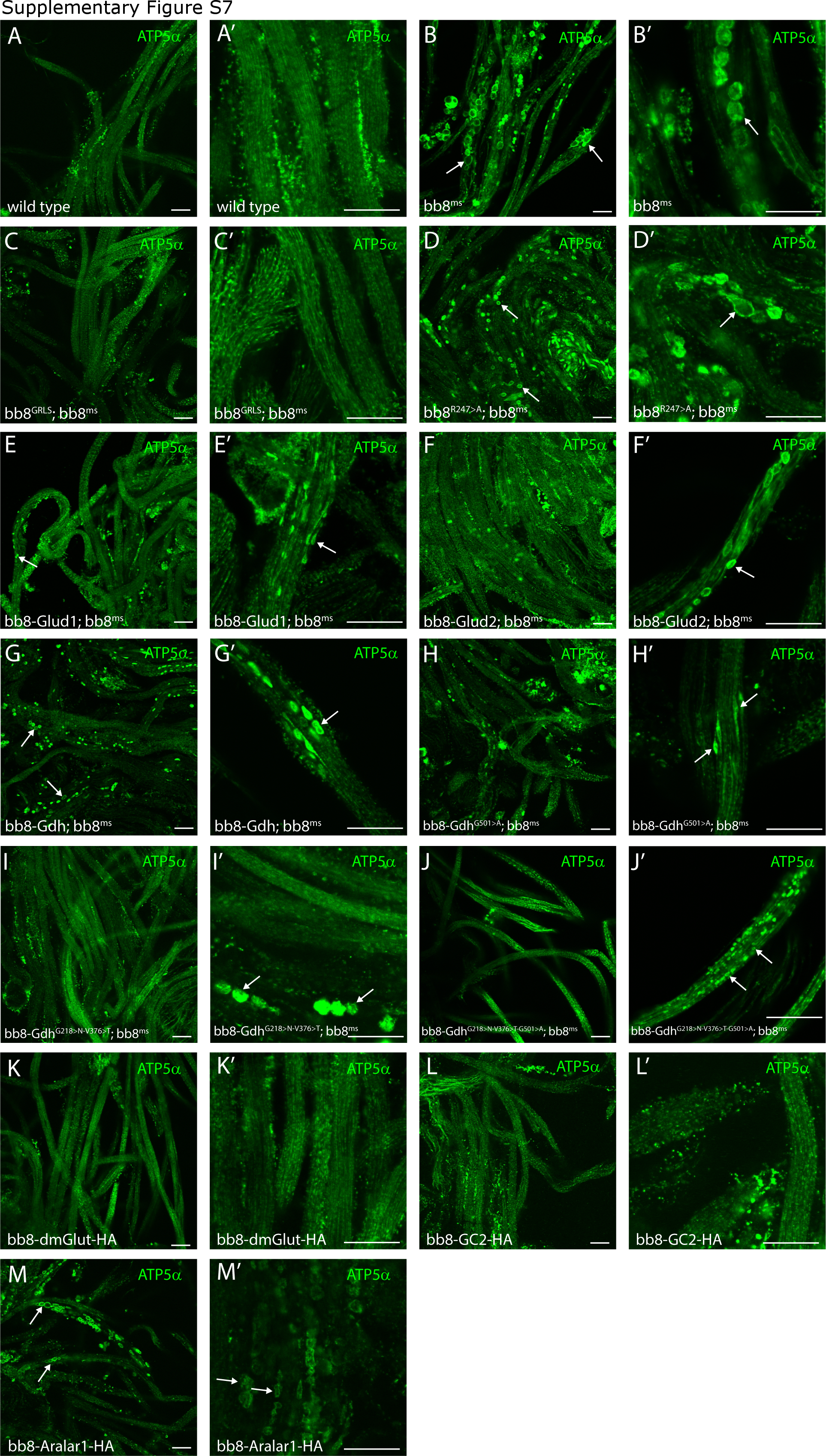

Supplement: Supplementary file 3 [file Presentation1.zip › Image 7.TIF]

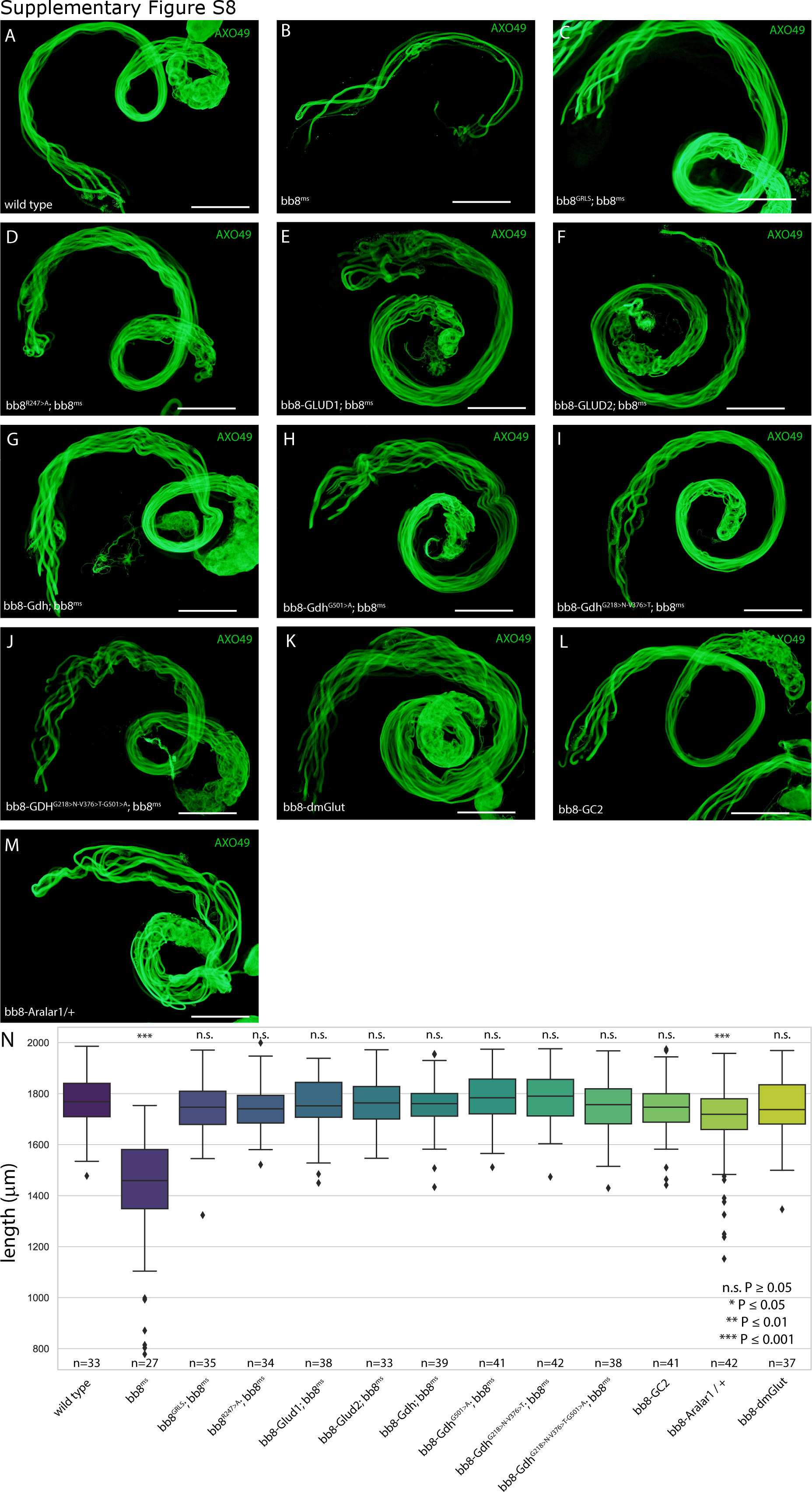

Supplement: Supplementary file 3 [file Presentation1.zip › Image 8.TIF]

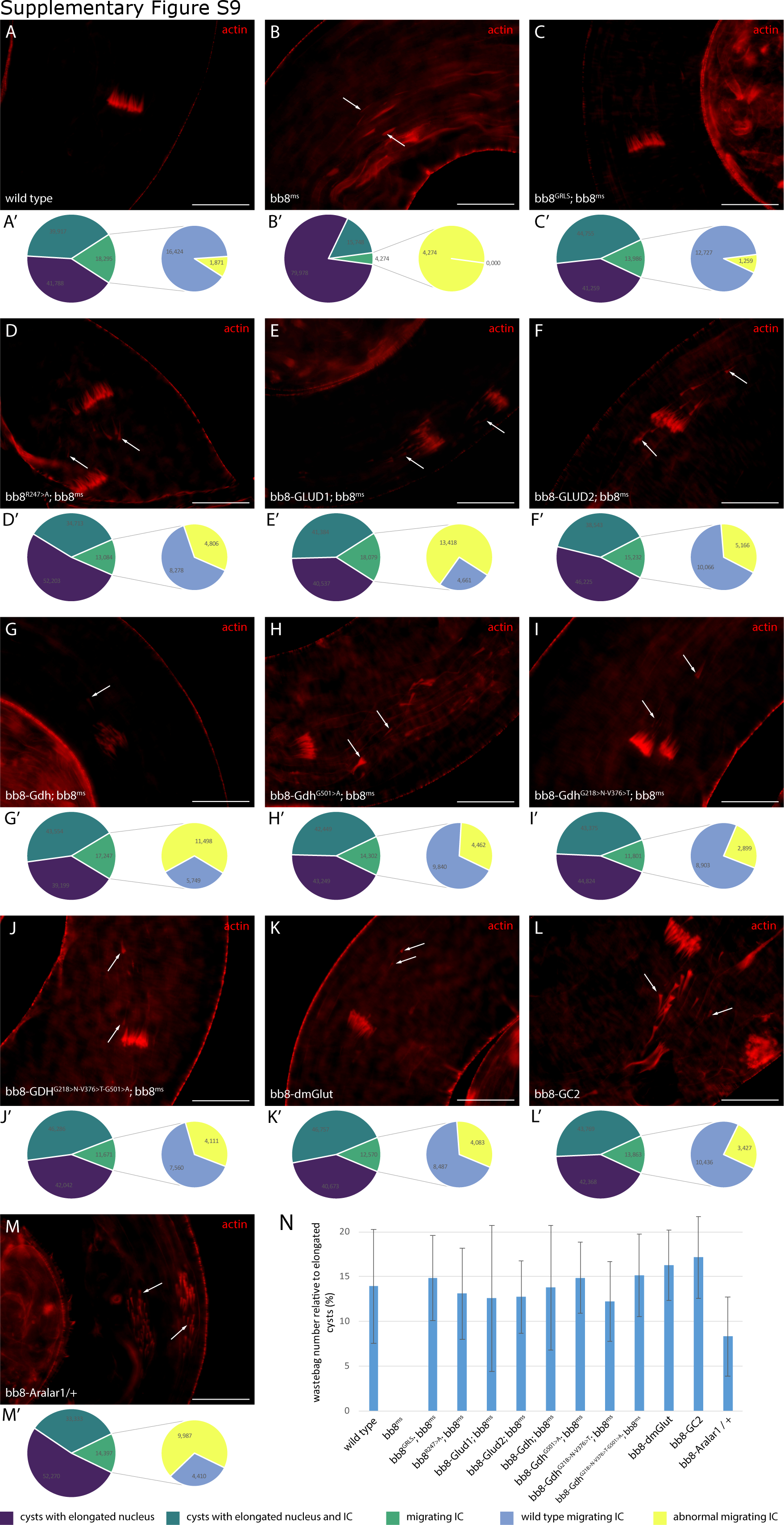

Supplement: Supplementary file 3 [file Presentation1.zip › Image 9.TIF]
