# Supplementary material for: A comparative analysis of fruit fly and human glutamate dehydrogenases in Drosophila melanogaster sperm development
Source: Front Cell Dev Biol. 2023 Nov 2;11:1281487. doi: 10.3389/fcell.2023.1281487 (PMC10652781; doi:10.3389/fcell.2023.1281487)
Supplement: Supplementary file 8 [file DataSheet1.docx]

**Fig S1 Molecular models, and organization of Glutamate dehydrogenases.** (A) The mammalian glutamate dehydrogenase forms homotrimers, which assemble into homohexamers. The hexamers could form filament-like structures. The structures presented correspond to the PDB model 1NQT of bovine glutamate dehydrogenase (B) InterEvDock3 (IED3) models of *Drosophila melanogaster* Gdh and Bb8 suggesting that their molecular structure and supramolecular organization could be similar to the bovine GDH.

**Fig S2.** **Clustal alignment of human and Drosophila glutamate dehydrogenases** Characteristic features are highlighted according to the legend

**Fig S3** **Phylogenetic tree of duplicated metazoan glutamate dehydrogenases** Larger taxonomic units highlighted by shading, coloured arrows point to different species.

**Fig S4 Functional site conservation, and position.** (A) Functional sites with highlighted conserved amino acids of human and drosophila GDH (B) Bb8 monomer molecule model (AlphaFold Q9VCN3-F1) with highlighted functional sites. In rows, we highlighted the same pocket in different angles/rotations (to visualize intramolecular position), while the column molecule models have an identical view (to visualize the relation of the pockets to each other).

**Fig S5** **Phasecontrast images of intact testes.** Yellow arrows show potential mitochondrial aberrations in the form of vacuole-like structures (in most cases white spherical objects- bubbles). Scale bars represent 200 μm.

**Fig S6 Confocal images of intact testes stained with MitotrackerRed.** Arrows highlight mitochondrial abnormalities. Mitotracker stains mitochondria with active membrane potential (megamitochondria often stain poorly). Scale bars represent 20 μm.

**Fig S7** **Confocal microscopic images of squashed testis immunostained with ATP5α antibody.** Arrows point to mitochondrial abnormalities. ATP5α antibody staining reveals structural abnormalities (good for visualization of megamitochondria). Scale bars represent 20 μm.

**Fig S8 Elongation phenotypes** (A-M) Fluorescent microscopic images of intact testes. Polyglycylated tubulin is visualized by AXO49 antibody. (N) Length measurements of AXO49 positive cysts. The number of testes used for measurements is represented above the genotype.

**Fig S9 Individualization phenotypes** (A-M) Fluorescent microscopic images of intact testes. Individualization complexes were visualized with phalloidin TexasRed. Arrows show disturbed, or malformed actin cones. Scale bars represent 50 μm. (A’-M’) Pie charts represent the distribution of individualization complexes between elongated cysts (25 testes were counted for every genotype), sub-charts represent the distribution of disturbed individualization complexes. (N) The number of waste bags relative to the elongated cysts.

**Fig S10 Comparison of known (bovine) GDH crystal structures to Drosophila Gdh models.** GDH could have multiple conformations, which complicates comparative analysis. (A) A comparison of opened and closed conformation of bovine GDH from cryo-EM experiments and crystal structure with the AlphaFold model of fly Gdh shows they have a similar structure but different conformations. Using Modeller we were able to create a *Drosophila melanogaster* Gdh model that is highly similar to the crystal structure of GDH (6DHN). (B) The same models are coloured by root-mean-square deviation (RMSD) of atomic positions between the two presented models.

**Supplementary Table 1** Table contains (A) Substitutive and non-substitutive alteration analyses of Drosophila melanogaster Gdh and Bb8 (B) Gdh and Bb8 substitution sites on sequences (C) Gdh and Bb8 functional pockets (D) InterEVDock3 scores and related template IDs. (E, F) SwissDock scores related to NADH binding to wild type and G218>N, V376>T mutant Gdh.

**Supplementary Table2** Table contains (A) the results of ancestry analyses of Bb8 like GDHs (B) the ancestral sequence of pockets.

**Supplementary Table3** Table contains data of amino acid conservation of Gdh, Bb8, Glud1 and GLUD2 further explanation is available on the Legend sheet.

**Supplementary Table4** Table contains the oligonucleotide sequences used for this study

**Supplementary video 1** GDH model transforming between open and closed form.
